# Identification of a unique temporal signature in blood and BAL associated with IPF progression

**DOI:** 10.1038/s41598-020-67956-w

**Published:** 2020-07-21

**Authors:** Katy C. Norman, David N. O’Dwyer, Margaret L. Salisbury, Katarina M. DiLillo, Vibha N. Lama, Meng Xia, Stephen J. Gurczynski, Eric S. White, Kevin R. Flaherty, Fernando J. Martinez, Susan Murray, Bethany B. Moore, Kelly B. Arnold

**Affiliations:** 1 Department of Biomedical Engineering, University of Michigan, Ann Arbor, MI 48109 USA; 2 Division of Pulmonary and Critical Care Medicine, Department of Internal Medicine, University of Michigan Medical School, Ann Arbor, MI USA; 3 Division of Allergy, Department of Medicine, Pulmonary and Critical Care Medicine, Vanderbilt University Medical Center, Nashville, TN USA; 4 Department of Biostatistics, School of Public Health, University of Michigan, Ann Arbor, MI USA; 5 Department of Internal Medicine, Weill Cornell School of Medicine, New York, NY USA; 6 Department of Microbiology and Immunology, University of Michigan Medical School, Ann Arbor, MI USA

**Keywords:** Proteome informatics, Respiratory tract diseases, Biomedical engineering, Inflammation

## Abstract

Idiopathic pulmonary fibrosis (IPF) is a progressive and heterogeneous interstitial lung disease of unknown origin with a low survival rate. There are few treatment options available due to the fact that mechanisms underlying disease progression are not well understood, likely because they arise from dysregulation of complex signaling networks spanning multiple tissue compartments. To better characterize these networks, we used systems-focused data-driven modeling approaches to identify cross-tissue compartment (blood and bronchoalveolar lavage) and temporal proteomic signatures that differentiated IPF progressors and non-progressors. Partial least squares discriminant analysis identified a signature of 54 baseline (week 0) blood and lung proteins that differentiated IPF progression status by the end of 80 weeks of follow-up with 100% cross-validation accuracy. Overall we observed heterogeneous protein expression patterns in progressors compared to more homogenous signatures in non-progressors, and found that non-progressors were enriched for proteomic processes involving regulation of the immune/defense response. We also identified a temporal signature of blood proteins that was significantly different at early and late progressor time points (*p* < 0.0001), but not present in non-progressors. Overall, this approach can be used to generate new hypothesis for mechanisms associated with IPF progression and could readily be translated to other complex and heterogeneous diseases.

## Introduction

Idiopathic pulmonary fibrosis (IPF) is a heterogeneous and irreversible interstitial pneumonia, with symptoms including progressive cough, shortness of breath, and ultimately respiratory failure, with a median survival of only 3–5 years post diagnosis^1^. The disease is believed to be caused by a dysregulated wound healing response to various epithelial injuries leading to fibrosis of the lung interstitium^1^. Two medications (nintedanib^2^ and pirfenidone^3^) are effective treatments for IPF; though neither can reverse the disease^4^. Thus, lung transplantation is currently the only option for a cure^5^, even though this procedure has the highest failure rate of all organ transplantation options (54% at 5 years^6^). Better understanding of mechanisms underpinning progression of pulmonary fibrosis could lead to improved outcomes via identification of new therapeutic targets.

To add to the complexity surrounding IPF, disease progression is also heterogeneous, with some individual patients experiencing long-term stability and others rapid loss of lung function. A number of longitudinal cohort studies have been created with the goal of better characterizing IPF pathobiology using proteomic measurements^7–10^. These efforts have identified individual proteins, including blood MMP-7^11,12^, CCL18^13^, and blood surfactant protein D^14,15^, as potential prognostic biomarkers. However, it has been difficult to replicate these findings across multiple cohorts^16,17^, especially when attempting to validate specific, prognostically-relevant cut-off concentrations^17,18^.

One potential explanation for failure to validate a specific prognostic biomarker is that disease progression is driven by dysregulated proteomic signaling networks rather than individual proteins. This hypothesis is supported by the multiple known actions of the two FDA-approved drugs that slow IPF progression, nintedanib^19^ and pirfenidone^19^. The use of quantitative approaches to capture individual proteins within large clinical “omics” data sets has become a useful way to find new proteins associated with disease progression. Groups of proteins associated with progression that were identified by these approaches were characterized by biologically relevant functions, such as involvement in the immune system^20–22^, tissue reorganization^20,21,23^, and epithelial cell function^23^. While these results have highlighted potential prognostic biomarkers and biological functions associated with IPF progression, many of the techniques used in these discoveries emphasize the additive significance of each protein’s individual ability to differentiate progression status but do not capture protein “signatures”, or take into account potential protein networks associated with progression. In addition, none of these large scale blood proteomics studies investigated quantitative proteomic relationships across other tissue compartments such as the lung.

Data-driven (“machine learning”) modeling approaches are able to integrate data across multiple tissue compartments and assays to identify signatures of factors that are associated with the disease state^24,25^. They serve as valuable tools for network inference by identifying co-varying factors that aid in generating new hypotheses for mechanisms of action based on protein interaction pathways rather that individual proteins. Once identified and validated, these signatures may be used for diagnostic or prognostic purposes, or for generating new hypotheses for future experimental work. We have previously used these approaches to successfully identify a blood protein signature that differentiated healthy and IPF patients with high accuracy^26^, as well as signatures based on blood and sputum proteins and blood cell markers that differentiated stable and exacerbated chronic obstructive pulmonary disease (COPD) patients^27^.

In this work, we applied data-driven modeling approaches to blood and bronchoalveolar lavage (BAL) samples from patients enrolled in the COMET (Correlating Outcomes With Biochemical Markers to Estimate Time-progression in Idiopathic Pulmonary Fibrosis) study to gain insight into cross-tissue compartment and temporal mechanisms of action associated with IPF progression. We identified a signature of blood and BAL proteins that differentiated IPF progressors and non-progressors with high accuracy. This signature indicated more heterogeneous progressor subgroups compared to non-progressors, and that proteins elevated in non-progressors were enriched for regulation of immune, defense and inflammatory responses. Lastly, using measurements across multiple time points we were able to identify signatures indicative of temporal changes in the blood of progressors that was not present in non-progressors. Overall these results provide insight into mechanisms of IPF progression that could be investigated further in follow-up murine studies.

## Results

### Only a small number of individual blood proteins are differentially expressed across IPF progressors and non-progressors

**Figure 1:**
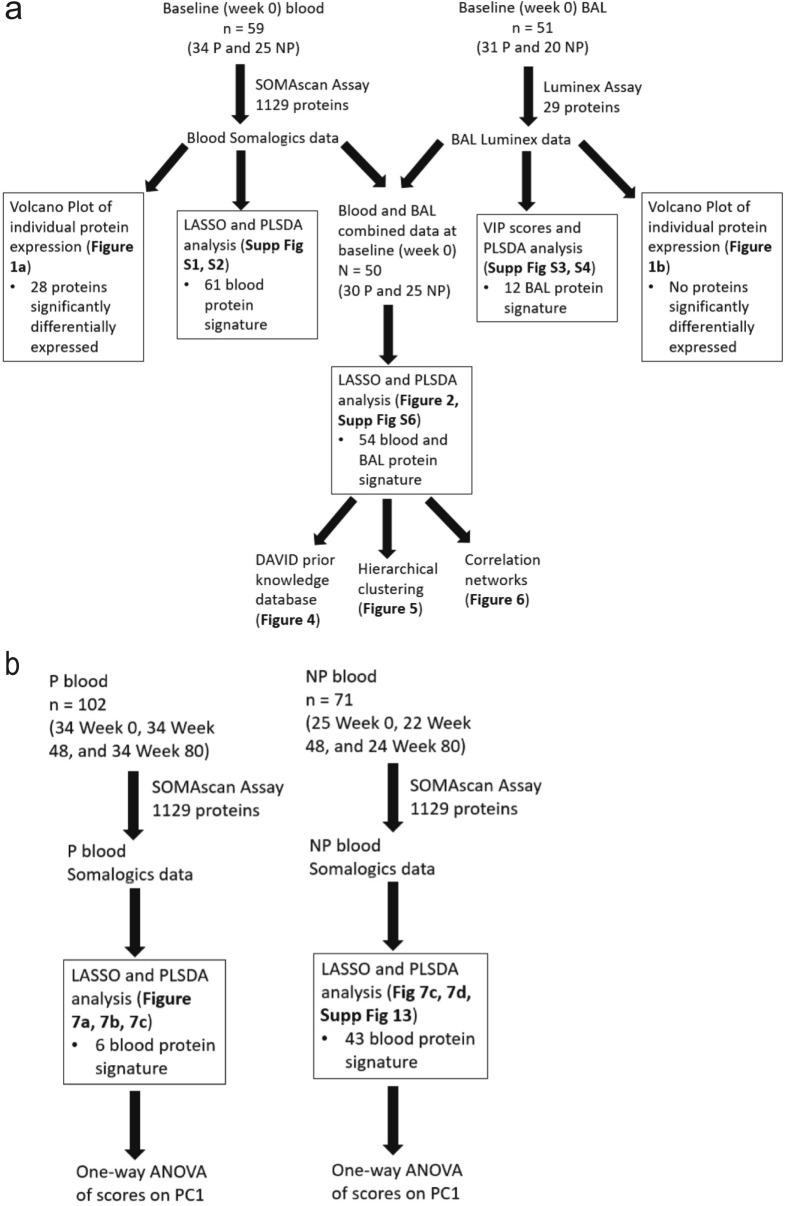
Schematic illustrating the number of samples and the computational tools used in analyses focusing on (**a**) comparing the inclusion of data from across multiple tissue compartments into data-driven models, and (**b**) comparing expression of proteins in the same patients over time. P, progressor; NP, non-progressor; BAL, bronchoalveolar lavage; LASSO, least absolute shrinkage and selection operator; PLSDA, partial least squares discriminant analysis; VIP, variable importance in projection; DAVID, database for annotation, visualization, and integrated discovery; PC1, principal component 1; Supp, supplemental.

We evaluated a subset of participants (n = 59) with an IPF diagnosis enrolled in the COMET IPF study. Participants were defined as progressors (n = 34) if at the end of the 80 week study they had experienced death, lung transplantation, an acute exacerbation of IPF (AE-IPF), or a drop in forced vital capacity (FVC) of > 10% or in diffusing capacity of the lung for carbon monoxide (DLCO) of > 15%^8^. Otherwise participants were defined as non-progressors (n = 25; demographics in Supplemental Table S1). Three blood draws from these 59 participants at week 0/baseline, 48 and 80 were used to measure the concentration of 1,129 proteins (enriched for inflammation and cancer involvement) with SOMAmer (slow off rate modified aptamer) technology (SomaLogic). One baseline (week 0) BAL sample was also collected from 51 individuals (31 progressors and 20 non-progressors; demographics in Supplemental Table S2; 50 of whom also had a baseline blood draw included in this analysis), and the concentration of 29 cytokines were measured with Luminex technology. There were no significant differences in demographic variables between the progressors and non-progressor groups, and all patients survived until the end of the 80-week study. Correlations in the periostin SOMAmer aptamer and ELISA measurement with these samples have previously been published^20^. To build on this, in Supplemental Table S3 we report significant Pearson’s correlations (all *p* < 0.03) between SOMAmer and ELISA concentrations for CCL22, CCL18, and CCL2, but not for IL-10 or CXCL12 (both *p* > 0.45). Our analysis pipeline is illustrated in Fig. 1: Fig. 1a focuses on analyses of baseline (week 0) expression of proteins in the blood and/or BAL samples of COMET patients, and Fig. 1b focuses on analyses of the temporal change in blood protein expression (week 0, week 48, and week 80). Within Fig. 1a, b, we have also annotated figures and supplemental figures associated with the results of each analysis.

**Figure 2:**
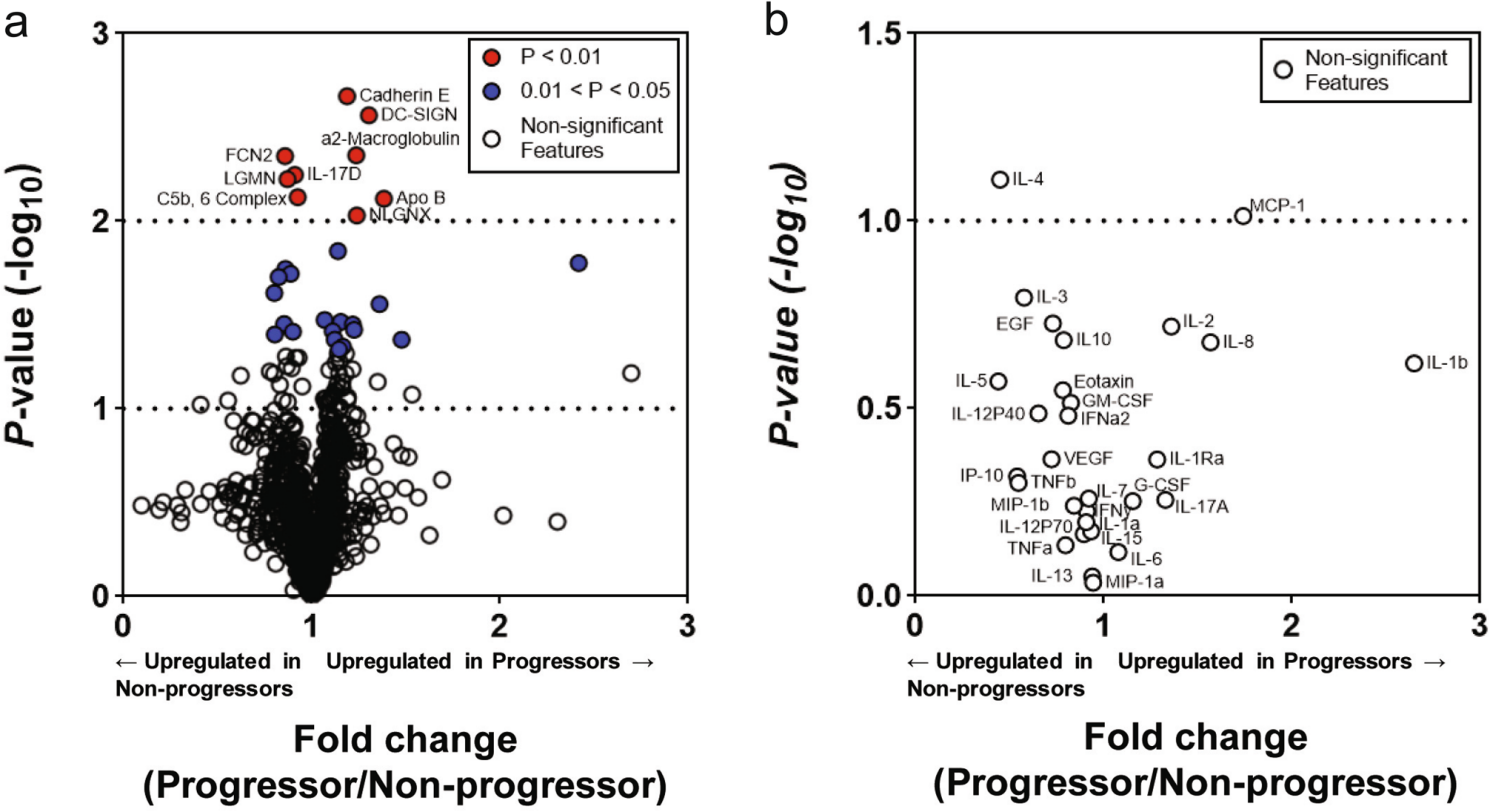
Volcano plot of blood (**a**) and BAL (**b**) proteins measured in COMET progressors and non-progressors. Proteins with a fold change greater than one are increased in progressors; fold changes less than one indicates elevation in non-progressors. Blue protein markers have a *p* value < 0.05 after a two-tailed, two-sample *t* test; red markers indicate *p* value < 0.01 after the same test. No blood or BAL proteins were significantly different between progressors and non-progressors after adjusting for multiple comparisons using the Bonferroni correction.

We first determined which of the measured baseline (week 0) 1,129 blood and 29 BAL proteins were individually differentially expressed between IPF progressors (n = 30) and non-progressors (n = 20; demographics of these 50 patients are found in Table 1). A two-sample t-test was applied to each protein expression in progressors and non-progressors and revealed that 28 blood proteins were significantly different across the two groups; 17 proteins were increased in the progressors (fold change greater than 1) (Fig. 2a; blue markers indicate a *p* < 0.05 and red indicate *p* < 0.01). The ten most significantly different blood proteins included E-Cadherin (cadherin E; fold change 1.19); DC-SIGN (CD209 antigen; fold change 1.30); a2-macroglobulin (fold change 1.24); ficolin-2 (FCN2; fold change 0.86); interleukin 17D (IL-17D; fold change 0.91); legumain (LGMN; fold change 0.87); C5b,6 complex (fold change 0.93); apolipoprotein B (ApoB; fold change 1.38); and neuroligin-4, X-linked (NLGNX; fold change 1.24). Except for TGM3 (protein-glutamine gamma-glutamyltransferase E; fold change of 2.47), all proteins had fold change values that ranged from 0.80 to 1.48. No BAL proteins were found significantly differentially expressed (Fig. 2b). No proteins in blood or BAL were significant after application of the Bonferroni correction for multiple comparisons.

**Table 1:** Demographic and lung function test descriptions from progressors and non-progressors whose baseline blood and BAL protein measurements were used in creating models based on the combination of blood and BAL proteins.

	Non-progressor (N = 20)	Progressor (N = 30)	*p* value
Age	62.43	65.36	0.2109
Sex (male)	16 (80%)	19 (63.3%)	0.2157
Number never smokers	6 (30%)	11 (36.7%)	0.6343
Number former smokers	13 (65%)	19 (63.3%)	0.9067
Number current smokers	1 (5%)	0	0.2243
FVC% predicted	66.88%	70.94%	0.4153
DLCO% predicted	45.78%	47.74%	0.626

### Data-driven analyses identify best signatures in single tissue compartments that differentiate IPF progression status

Due to the low number of significantly differentially expressed proteins in the univariate analysis, we next determined whether data-driven modeling techniques could identify signatures of proteins from single tissue compartments that differentiated IPF progressors and non-progressors. Our analysis pipeline that focused on baseline (week 0) expression of proteins in the blood and/or BAL samples of COMET patients is visualized in Fig. 1a. We used the least absolute shrinkage and selection operator (LASSO^28^) as a feature selection tool to identify a signature of baseline (week 0) blood proteins that would best differentiate COMET participants based on progression status at 80 weeks. For every LASSO model in this analysis, k-fold cross-validation (k = 10; see Methods) was performed to prevent over-fitting. Feature selection was accomplished in the BAL proteins through the use of variable importance in projection (VIP) scores. We then employed partial least squares discriminant analysis (PLSDA^29^) in order to visualize the separation power of the identified signatures. By highlighting co-varying relationships within protein signatures, PLSDA aids in generating new hypotheses about proteomic pathways associated with each group. For every PLSDA model in this analysis, we calculated calibration and k-fold cross-validation accuracy (k = 10) to use as metrics of model performance for comparing PLSDA models generated from data in different tissue compartments (see Methods). LASSO identified a signature of 61 blood proteins that differentiated 25 non-progressors and 34 progressors (demographics in Supplemental Table S1); a PLSDA model based on this signature had 100% calibration and 96.53% cross-validation accuracy (Supplemental Figure S1a and S1b; ROC curves in Supplemental Figure S2). The PLSDA model based on 12 VIP-selected baseline (week 0) BAL proteins differentiated 20 non-progressors and 31 progressors (demographics in Supplemental Table S2) with 78.55% calibration and 67.82% cross-validation accuracy (Supplemental Figure S3a and S3b; ROC curves in Supplemental Figure S4). Although these models performed with moderate to excellent accuracy, we wanted to explore the unique biological insight that might be gained from a model based on the combination of the data from the two tissue compartments.

### Cross-tissue compartment signature differentiates COMET participants based on progression status

**Figure 3:**
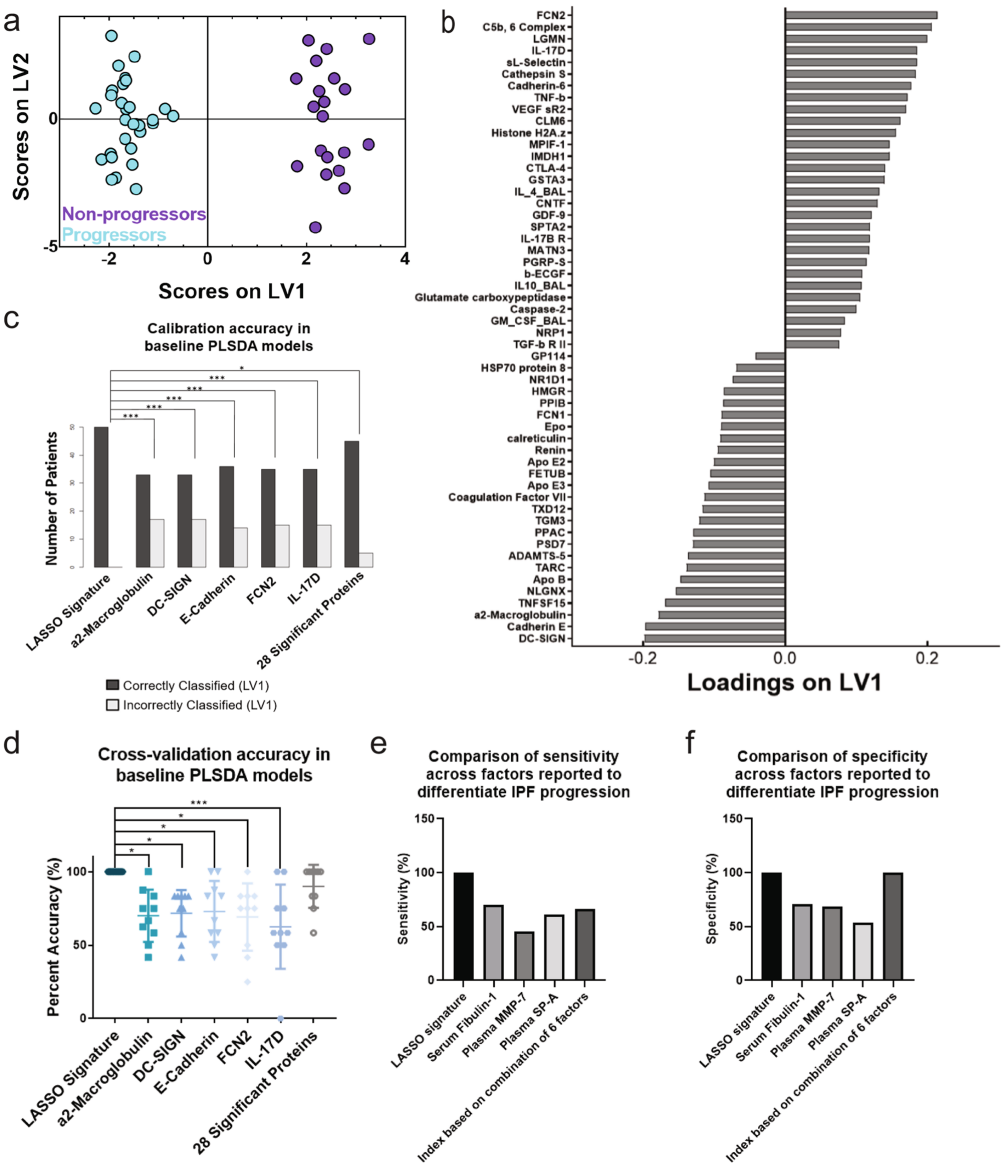
The LASSO-identified signature based on blood and BAL proteins separated progressors and non-progressors with high accuracy and significantly outperformed analyses based on individual factors. (**a**) PLSDA scores plot based on blood and BAL proteins highlights strong differentiation between progressors (cyan) and non-progressors (purple); the model separated the two groups with 100% cross-validation and calibration accuracy. (**b**) The loadings on latent variable 1 (LV1) captured 8.75% of the total variance in the data, with negatively loaded proteins being comparatively increased in progressors and positively loaded proteins being comparatively reduced. (**c**) Comparison of the calibration accuracies between analyses based on data-driven signatures and univariate factors. The LASSO-selected PLSDA model based on blood and BAL proteins had significantly higher calibration accuracy than all analyses based on single proteins and a model based on the collection of all 28 significantly different proteins identified in Fig. 1 (Cochran’s Q test with McNemar’s post hoc test; *p < 0.05; ***p < 0.001). (**d**) Comparison of cross-validation accuracies between analyses based on data-driven signatures and univariate factors. The LASSO-selected PLSDA model based on blood and BAL proteins had significantly higher cross-validation accuracy than all analyses based on single proteins and trended towards better cross-validation accuracy than a model based on the 28 proteins identified in Fig. 1 (one-way ANOVA with Tukey’s post hoc test; *p < 0.05; ***p < 0.001). (**e**) Comparison of sensitivity between the LASSO-selected PLSDA model based on blood and BAL proteins and previously published models of IPF progression (serum fibulin-1^30^, plasma MMP-7^31^, plasma SP-A^31^, and an additive combination of blood factors^20^). (**f**) Comparison of specificity between the LASSO-selected PLSDA model based on blood and BAL proteins and previously published models of IPF progression.

We combined measurements of the 1,129 blood proteins and 29 BAL proteins from baseline samples to identify a cross-tissue compartment signature of co-varying proteins associated with progression. LASSO identified a signature of 54 baseline (week 0) proteins (51 in blood and 3 in BAL) that best separated progressors and non-progressors (comparison of protein signature expression in progressors and non-progressors can be found in Supplemental Figure S5). A PLSDA model based on this signature classified the two groups with 100% cross-validation and calibration accuracy (Fig. 3a), with 100% sensitivity and specificity for each group (ROC curves in Supplemental Fig. 6) and with positive and negative predictive values of 100%. Latent variable 1 (LV1) differentiated progressors (negative scores on LV1) from non-progressors (positive scores on LV1) (Fig. 3b). Interestingly, we did not find significant Pearson’s correlation between the scores on LV1 in this signature and the concentration of KL-6 (r = 0.15, *p* = 0.31), MMP7 (r = − 0.08, *p* = 0.60), or CCL18 (r = 0.04, *p* = 0.77), which were other previously identified individual biomarkers of progression. However, we did see a significant correlation between the LV1 scores and the change in FVC percent predicted over the 80 weeks of the study (r = 0.534, *p* = 0.00011, Pearson’s correlation coefficient).

We compared this model to cross-validated analyses based on single significant proteins identified in the volcano plot, as well as a cross-validated PLSDA model based on the collection of the 28 differentially expressed blood proteins in the volcano plot (ROC curves for last model shown in Supplemental Figure S7). The model based on the LASSO-identified signature had significantly higher calibration accuracy than all of the analyses based on the individual proteins and the collection of the differentially expressed proteins (Fig. 3c; Cochran’s Q test with McNemar’s post hoc test). In terms of cross-validation accuracy, the LASSO-identified model also significantly outperformed analyses based on all of the individual proteins, and trended towards outperforming the model based on the collection of the 28 differentially expressed proteins (Fig. 3d; one-way ANOVA).

We also compared this model to other previously published single markers and combinations of markers that were shown to differentiate IPF progression status. The model based on our signature had 100% sensitivity and specificity, which outperformed previously published models that predicted IPF progression based on single factors (serum fibulin-1, 70% sensitivity and 71% specificity^30^; plasma MMP-7, 45.3% sensitivity and 68.5% specificity^31^; and plasma SP-A, 60.9% sensitivity and 53.9% specificity^31^), as well as a previously published model based on an additive combination of blood factors, where a score of ≥ 7 on the created index had a 66% sensitivity and 100% specificity for progression^20^ (Fig. 3e, f).

We next sought to determine if the PLSDA model based on the combination of blood and BAL proteins was a better classifier than models based on signatures of blood or BAL proteins alone. The model based on blood proteins alone and the model based on blood and BAL proteins combined had significantly higher calibration accuracy than the model based on BAL proteins alone (Supplemental Figure S8a, *p* = 0.0016 for marked comparisons; Cochran’s Q test with McNemar’s post hoc test applied to calibration accuracy of patients that were included in all three models). McNemar’s post hoc test could not be applied when comparing the calibration accuracies of the blood protein model and the combination model because all patients were classified correctly in both models. When comparing cross-validation accuracies across the three models, again the model based only on BAL proteins performed significantly worse than the blood protein model and the combination model (Supplemental Figure S8b, *p* = 0.0001 for the blood protein vs. BAL protein model comparison and *p* < 0.0001 for the BAL protein vs. combination model comparison, one-way ANOVA with Tukey’s post hoc test applied to cross-validation accuracy based on all patients in all three model).

One reason the model based on BAL proteins had lower calibration and cross-validation accuracies might involve the high number of measured blood versus BAL proteins (1,129 blood proteins vs. 29 BAL proteins). To investigate the potential effect of signature size on model accuracy, we created two new PLSDA models: one based on the top 12 loaded features of the blood signature; and the other based on the top 11 loaded proteins (all of which were blood proteins) and the top loaded BAL protein in the combination signature, for a total of 12 proteins in this shortened combination signature. When comparing the calibration accuracies of these models with the same signature size, there was no significant difference between the performance of the BAL protein model and the shortened blood protein model (*p* = 0.78, Cochran’s Q test with McNemar’s post hoc test). However, the calibration accuracy of the shortened combination model trended towards being significantly better than both of the BAL protein and the shortened blood protein models (*p* = 0.052 for both comparisons, Cochran’s Q test with McNemar’s post hoc test, Supplemental Figure S9a). There were no significant differences in cross-validation accuracy across any of the models, but again the shortened combination model trended towards significantly outperforming the BAL protein model (*p* = 0.12, one-way ANOVA with Tukey’s post hoc test; Supplemental Figure S9b). Overall this suggests that the model based on blood proteins alone may have performed well due to the large panel of proteins measured, though the combination model still trends towards being significantly better than the BAL model even when the signature is shortened. We next explored the biological significance of the combination signature.

### Non-progressors have enriched regulation of immune and defense responses, and protein expression patterns suggest more heterogeneity in progressors

**Figure 4:**
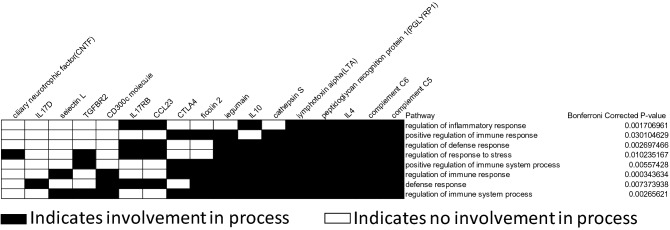
DAVID enrichment analysis of the blood and BAL LASSO-identified proteins that were comparatively elevated in the non-progressor group in the PLSDA loadings plot showed enrichment for pathways involved in the regulation of the inflammatory, defense, and immune responses after application of the Bonferroni correction (enrichment score 4.83). Black squares indicate protein involvement in a particular pathway, while white squares indicate non-involvement.

The database for annotation, visualization and integrated discovery (DAVID^32^) determined the proteins that were comparatively increased in the non-progressors in the LASSO-identified signature based on blood and BAL proteins were significantly enriched for processes involving immune and defense response regulation [Fig. 4, enrichment score (ES) 4.83]. Other functions enriched in non-progressors included cell signaling and regulation of basic cell processes (Supplemental Figure S10a, ES 2.57), and regulation of inflammatory, defense, and immune responses (Supplemental Figure S10b, ES 2.50). DAVID identified that proteins that were comparatively increased in progressors were only enriched for stress response regulation (Supplemental Figure S11, ES 2.05).

**Figure 5:**
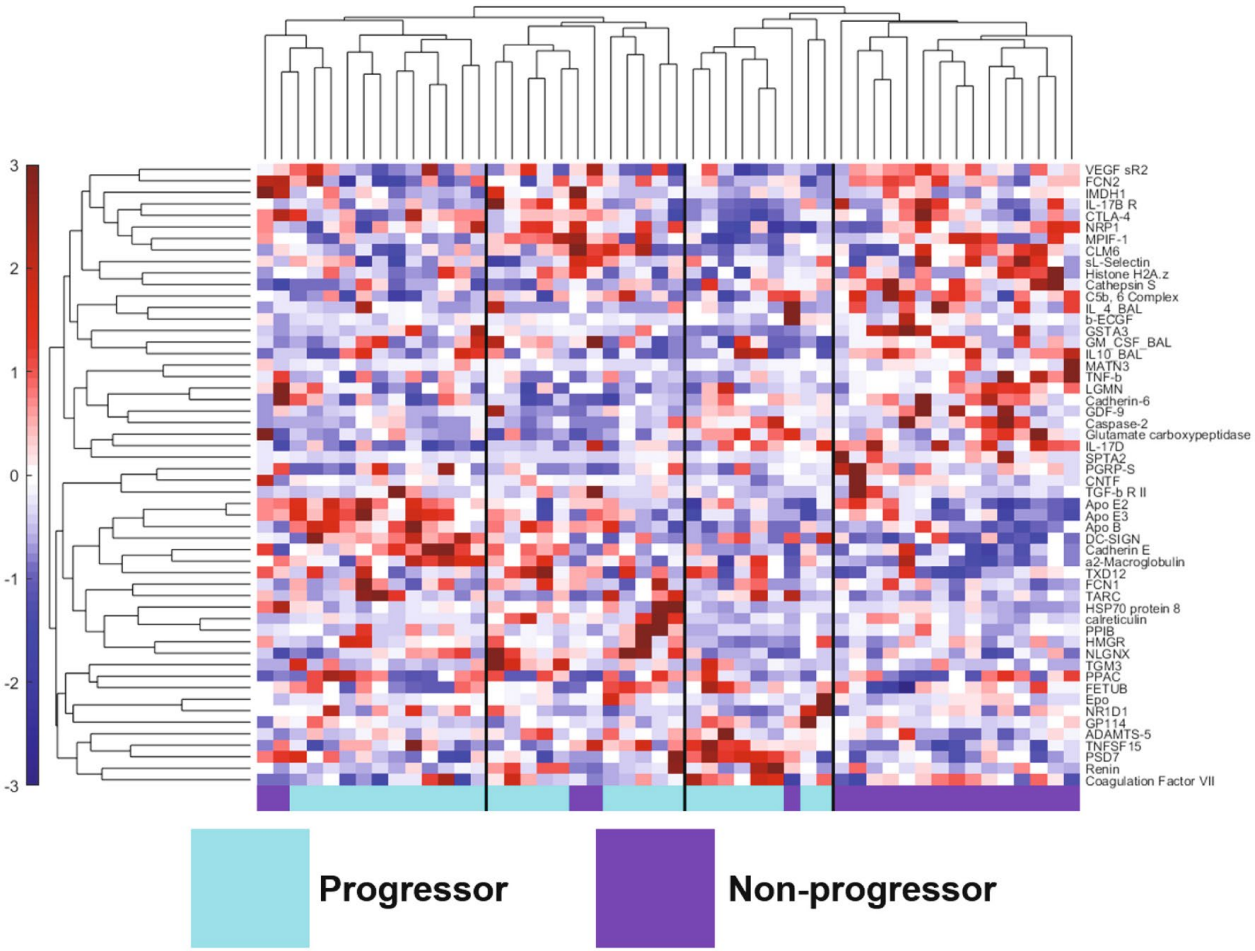
Hierarchical clustering of the COMET IPF patients by the LASSO-identified blood and BAL protein signature highlights a single group of non-progressors (purple) and three groups of progressors (cyan) with distinct expression levels of various proteins in the signature. Only 5 out of the 50 patients were misclassified. Protein expression level is shown in the color scale on the left of the figure, with red indicating higher concentration compared to the mean, and blue lower concentration compared to the mean.

We next used hierarchical clustering to visualize the individual expression of the proteins in the blood and BAL protein signature across all the patients. We saw clear clustering of the two groups, with one cluster composed only of non-progressors and three clusters that were mostly progressors (Fig. 5). Only 5 non-progressors were misclassified out of 50 patients total (90% classification accuracy; 100% sensitivity and 75% specificity for identification of progressors). There were minor differences in classification accuracy of the PLSDA model and hierarchical cluster, likely due to underlying algorithmic differences associated with unsupervised identification of groups via the Pearson distance metric (hierarchical clustering) vs. supervised identification of groups based on maximized covariance in protein expression (PLSDA). Interestingly, there was heterogeneity within the progressor cluster, which was characterized by expression of different proteins. One of the progressor clusters had many apolipoproteins overexpressed compared to the mean (apolipoproteins E2, E3, and B), as well as cadherin E and DC-SIGN. Other progressors had high expression levels of proteins that were also highly expressed in the first group of progressors (apolipoproteins E3 and B, and cadherin E), as well as proteins that were expressed highly in the non-progressor cluster (CTLA-4, MPIF-1/CCL23, and IL-17B receptor). The third group of progressors was characterized by high expression of TNFSF15 (also known as vascular endothelial growth inhibitor) and PSD7 (26S proteasome non-ATPase regulatory subunit 7). The presence of the three progressor groups in the hierarchical cluster may suggest heterogeneity among progressors compared to relative homogeneity among non-progressors, however based on the small sample size in this data it is not possible to determine whether these groups arise from other co-variates and/or random effects. We did evaluate whether any of the progressor clusters could be explained by other clinical and radiological variables collected during the COMET study, including progression metric (e.g. through AE-IPF or a > 10% drop in FVC, etc.), smoking status, each participant’s genotyping at the MUC5B rs35705950 and the TOLLIP rs5743890 SNPs, and the presence of ground glass and honeycombing in their baseline CT scan. We did not find any apparent clustering by any of these other variables (Supplemental Figures S12a–h).

### Non-progressors exhibit fewer and stronger protein correlations at baseline (week 0) than progressors

**Figure 6:**
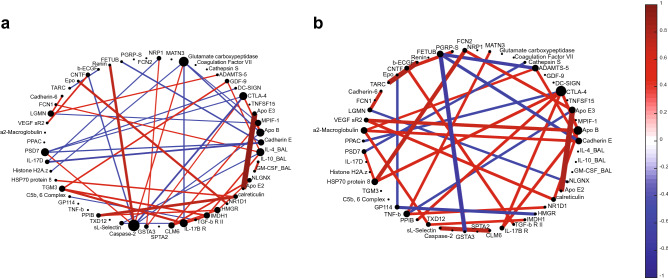
Protein correlation networks of the LASSO-identified blood and BAL protein signature present in progressors (**a**) and non-progressors (**b**) suggest that non-progressors have a higher degree of control over their proteomic networks than progressors. A lineconnecting two proteins indicates the presence of a significant (*p* < 0.05) correlation, as calculated by Pearson’s correlation coefficient. Brighter and thicker lines indicate stronger, more significant correlations, respectively. The value of the correlation coefficient for both networks is displayed in the color bar scale on the right, with red indicating a positive relationship and blue a negative relationship.

Interestingly, when we used correlation networks to explore relationships between proteins in the LASSO-identified signature based on blood and BAL proteins, we found the network based on signature expression levels in progressors had a larger number of overall weaker correlations than the network based on non-progressors. The protein correlation network based on progressors’ protein expression (Fig. 6a) contained seven proteins with at least four significant correlations to other proteins. We speculate that the presence of numerous proteins with high numbers of significant correlations (i.e. hub proteins) may suggest a network with multiple potential drivers, especially when compared to the correlation network based on non-progressors’ protein expression (Fig. 6b), which only contained two proteins with four or more significant correlations. Blood caspase-2, CTLA-4, and ApoB, and BAL IL-4 were hub proteins in the progressor network, while blood CTLA-4 and ApoB were the hub proteins in non-progressors. When comparing the two networks, it was clear that there were fewer (45 correlations vs 33 in the non-progressor network), but significantly stronger (higher absolute value; *p* = 0.0002, two-sample t-test) correlations present in the non-progressor network.

### Trajectory principal component analysis (PCA) identified significant differences in the temporal signature of progressors that were not present non-progressors

**Figure 7:**
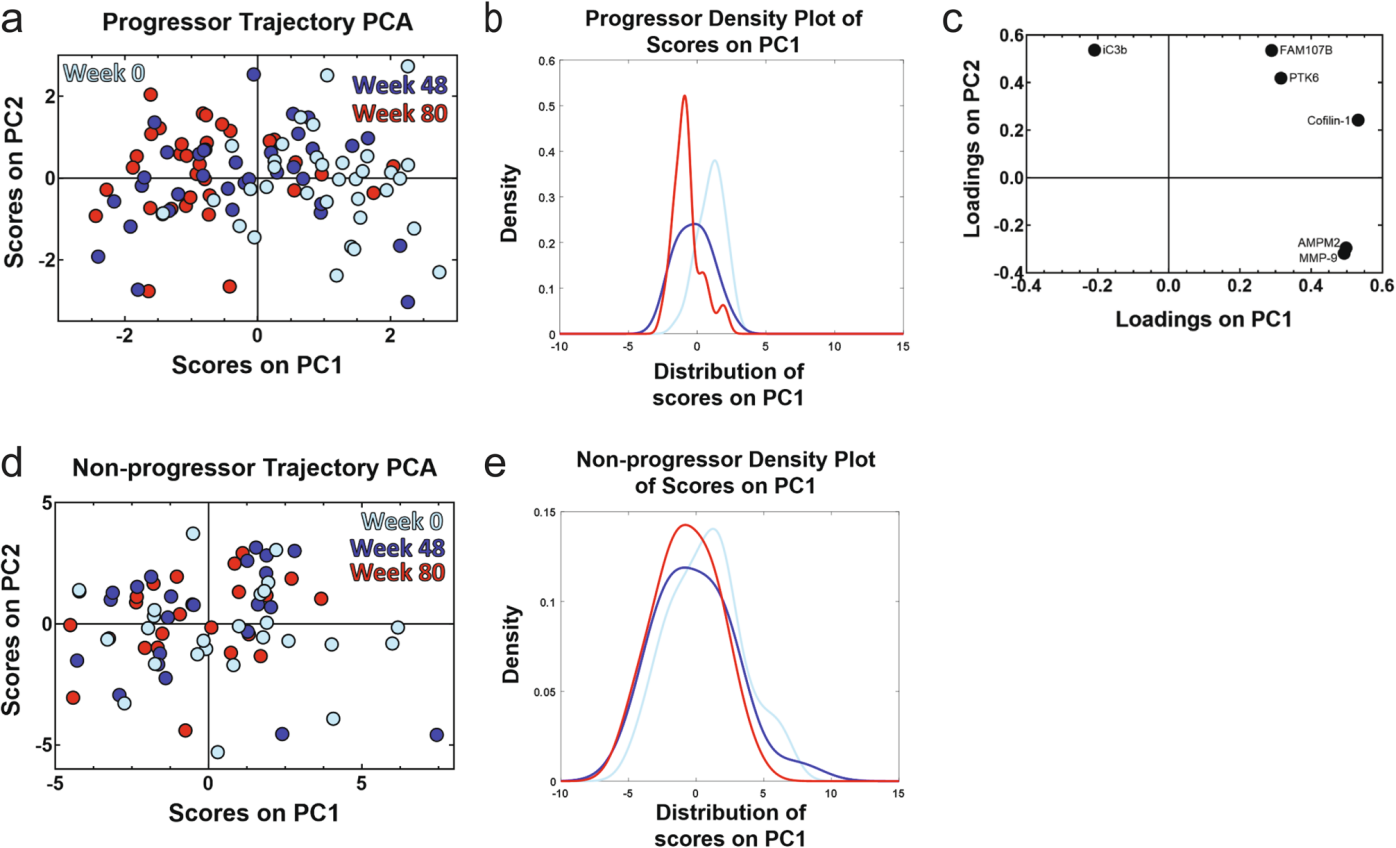
Trajectory PCA highlights changes in blood protein expression over time in progressors that is not seen in non-progressors. (**a**) A trajectory PCA model based on three time points of progressor blood protein measurements highlights the change in protein expression patterns over time in IPF progressors. The week 0 scores on principal component 1 (PC1) were found to be significantly different from both the week 48 scores (*p* < 0.001) and the week 80 scores (*p* < 0.001) by one-way ANOVA with Tukey’s post hoc test. The week 48 and week 80 scores were not found to be significantly different from one another by the same test (*p* = 0.16). (**b**) The kernel density plot of the scores on PC1 provides another way of viewing the differences in the scores distribution of across all three time points of progressors. (**c**) The LASSO-identified signature separates the three time points of progressor measurements while capturing 49.95% of the natural variance in the data across the first two principal components. (**d**) A trajectory PCA model based on three time points of non-progressor protein measurements does not show clear separation across the three time points. None of the scores on PC1 of the three time points were significantly different from each other after one-way ANOVA with Tukey’s post hoc test (all *p* > 0.05). (**e**) The kernel density plot of the scores on PC1 highlights the overlapping of the scores on PC1 from the three time points of non-progressors.

Finally, we found a time-dependent shift in protein expression in progressors that was not present in non-progressors. Our temporal analysis pipeline is illustrated in Fig. 1b. We used LASSO and associated cross-validation to identify signatures that differentiated three time points of blood protein expression (week 0/baseline, week 48, and week 80) within progressors and non-progressors. We then created trajectory principal component analysis (PCA) models^33^ based on these signatures to judge temporal separation. The trajectory PCA based on progressor measurements found significant differences in the temporal signature for week 0 and week 80 measurements, with week 48 time points falling in between the other two (Fig. 7a). A one-way ANOVA with Tukey’s post hoc test found that week 0 progressor scores on principal component 1 (PC1) were significantly different than scores from week 48 and week 80 (*p* < 0.0001 for both comparisons). We also created a kernel density plot based on the progressor scores on PC1 to further illustrate the differences in the spread of scores between week 0 and week 80 (Fig. 7b). The accompanying loadings plot (Fig. 7c) indicated a relative increased expression of inactivated complement C3b (iC3b) compared to matrix metalloproteinase 9 (MMP-9), methionine aminopeptidase 2 (AMPM2), cofilin-1, protein tyrosine kinase 6 (PTK6), and protein FAM107B at week 80, but relative increase of MMP-9, AMPM2, cofilin-1, PTK6, and protein FAM107B compared to iC3b at week 0. In contrast, a trajectory PCA model for non-progressors (Fig. 7d) and a one-way ANOVA with Tukey’s post hoc test indicated there were no significant differences in PC1 scores across the three time points (*p* > 0.05 for all comparisons; loadings plot shown in Supplemental Figure S13). The kernel distribution plot of the non-progressors’ scores on PC1 highlights how all three time points are spread out among the same range of scores (Fig. 7f).

## Discussion

In this work we have identified cross-tissue compartment and temporal proteomic signatures that highlight differences between IPF progressors and non-progressors and generate new hypotheses for potential mechanisms of IPF progression. We discovered a multivariate signature based on proteins from the blood and lung tissue compartments that differentiated IPF progressors and non-progressors with 100% cross-validation and calibration accuracy and 100% sensitivity and specificity in a PLSDA model. This signature performed significantly better than analyses based on single proteins and a signature of BAL proteins. Through the use of other computational tools, we found that non-progressors were enriched for regulation of immune regulatory processes, and that the proteome of progressors had significantly fewer and weaker correlations than that of non-progressors. Using data from across multiple time points, we were able to identify significant proteomic differences in IPF progressors between week 0 and week 80 measurements that were not present in non-progressors. These results illustrate the value of data-driven modeling approaches for integrating measurements over different tissue compartments and experimental assays, and suggested potential prognostic signatures for progressive IPF for future validation.

The combined use of LASSO with PLSDA allowed us to find small signatures out of hundreds of proteins that were able to accurately differentiate clinical groups of interest. PLSDA and LASSO were able to incorporate data from multiple tissue compartments and assays in the same model to enable a more systemic understanding of IPF progression. The signature of co-varying blood and BAL proteins that we reported has the highest cross-validation and calibration accuracy compared to models based on single proteins, and either outperformed or matched the sensitivity and specificity of previously reported markers of IPF progression. Evaluating signature components allowed for further investigation of potential proteomic relationships and pathways associated with progression. Our identified signature was enriched for processes involving immune system regulation in non-progressors, which echoes results from other studies^20,22,23^, and also included 4 of the 6 proteins previously identified in the COMET cohort as an index of IPF progression^20^. The complement cascade has also previously been associated with IPF disease severity^22^. Interestingly, our identified signature did not include MMP-7, which has been linked to IPF progression in several other studies^21,23,34^, though some proteins in our signature did have proteolytic function (legumain, PSD7).

There were several limitations associated with this study. While we were able to integrate SOMAmer- and Luminex-based measurements in our models, the SOMAlogic^©^ platform measured many more proteins than the Luminex platform, potentially biasing results toward blood measurements and toward the functions of the 29 BAL cytokines measured with Luminex. Larger (in the case of BAL proteins) and less directed screens of blood and BAL proteins in future experiments may uncover more unbiased signatures. Another consideration is that the aptamer measurements do not always significantly correlate with ELISA concentrations, which could be due to different actions and binding sites of aptamers vs. antibodies. All subjects in the COMET study lived through the study end date, which means that our presented hypotheses might not representative of end-stage IPF patients. Although the model based on both blood and BAL proteins was found to be the most accurate at differentiating IPF progression status, this model would not currently be useful as a prognostic test due to: (1) challenges associated with obtaining BAL measurements; and (2) the large number of proteins currently in the signatures. However, because our model is able to investigate covariation in protein expression across tissue compartments, we do believe that the analysis is useful for generating new insight into potential systemic, proteomic relationships associated with IPF progression. The blood protein signature identified here holds more promise as a prognostic signature (cross-validation accuracy of 96% was only moderately lower than the combined model), however would still require reduction in the number of proteins before it would be useful. Furthermore, development of a true prognostic signature for clinical use would require validation in new, larger cohorts. To our knowledge there is currently no appropriate validation cohort available, and the SOMAmer platform is no longer accessible for academic use. Therefore, we are unable to confirm the diagnostic or prognostic merit in any of the identified signatures. We did employ cross-validation which suggests that future validation of prognostic biomarkers could be valuable.

We identified signatures in our study to investigate potential mechanistic differences between IPF progressors and non-progressors, we found several emerging trends. A prior knowledge database (DAVID) indicated that significantly enriched processes in non-progressors involved regulation of immune or defense system responses, suggesting that this regulation is potentially lacking or deficient in progressors. We speculate that the idea that non-progressors have better control of proteomic processes was also reflected in the protein correlation networks, where non-progressors had fewer hub proteins and fewer significant correlations present, but these correlations were significantly stronger than those in the progressor network. We hypothesize this indicates a more stable protein network in non-progressors that would be difficult to perturb. Stronger correlations could also indicate that non-progressors have finer control over the expression of these proteins, suggesting that the biological pathways these proteins are involved in are less dysregulated than they are in progressors. Additional experimental analysis would be needed to confirm these ideas.

IPF progressors were characterized by more heterogeneous proteomic expression across tissue compartments. Heterogeneity was suggested by both the correlation network (the large number of significant but weak correlations present in progressors), and also in the hierarchical cluster, which exhibited three progressor clusters that were characterized by unique expression patterns of proteins. We speculate that this may suggest potential subgroups (endotypes) present within the progressors, however this study did not have the power to eliminate the effects of other co-variates or random influence. One progressor cluster showed increased expression of many apolipoproteins, in addition to DC-SIGN, E-cadherin, ficolin-1, and other proteins. Intriguingly, another cluster of progressors exhibited increased expression of both proteins that were also highly expressed in the non-progressor cluster and proteins that were highly expressed in another progressor cluster. We investigated this group of progressors but did not find a significant difference in the time from COMET enrollment to date of progressive event between this group and the other two groups of progressors identified in the hierarchical cluster. Unsupervised analytical and clustering techniques could be used in other larger studies to better characterize and confirm potential endotypes of IPF progressors.

Intriguingly, proteins from the complement system were signature components in both the temporal-focused and in the tissue compartment analyses. We observed that progressors at later time points (48 or 80 weeks post-baseline) were characterized by comparatively increased expression of iC3b compared to other proteins in the signature. iC3b plays a critical role in pathogen binding and clearance, and also regulates other functions including phagocytosis and IL-12 secretion^35,36^. To our knowledge there have been no studies directly focused on IPF and iC3b, but complement 3 (C3)’s involvement in IPF has been previously studied, with *C3* gene expression reported to be higher in the lungs of IPF patients vs. those of healthy controls^37^. Likewise C3 deficient mice exhibited reduced lung injury after exposure to bleomycin than their wild type counterparts^37^, and depletion of the serum complement system inhibited bleomycin-induced lung collagen deposition in rats^38^. Although these studies investigated C3 expression and fibrosis, in our data progressor iC3b expression was positively and significantly correlated with progressor C3 expression over all time points (Pearson’s correlation coefficient, ρ = 0.52, p-value = 2.1*10^−8^), suggesting that changes in iC3b expression levels reflect similar changes in C3 concentration. Although appearances of iC3b in identified signatures suggest an association with IPF progression, future experimental and clinical studies would be needed to confirm any mechanistic role.

In conclusion, we were able to use systems-focused, data-driven modeling approaches to identify temporal and cross-tissue compartment proteomic signatures that led to increased insight into mechanisms associated with IPF progression. Overall, this work highlighted the ability of quantitative, systems-focused analytical techniques to aid in generating novel hypotheses for proteomic mechanisms associated with IPF progression. We envision these approaches could be easily applied to integrate spatiotemporal data in clinical samples from other diseases that have a progressive and/or heterogeneous patient population.

## Methods

### Ethical approval statement

All clinical investigations were conducted according to the Declaration of Helsinki. The human study protocol was approved by the institutional review board of all participating centers and methods were carried out in accordance with the relevant guidelines and regulations (University of California Los Angeles, Los Angeles, CA, United States; University of California, San Francisco, San Francisco, CA, United States; National Jewish Medical and Research Center, Denver, CO, United States; University of Chicago, Chicago, IL, United States; University of Michigan Ann Arbor, MI, United States; Cleveland Clinic Foundation, Cleveland, OH, United States; Temple University, Philadelphia, PA, United States; Brown University, Providence, RI, United States; Vanderbilt University, Nashville, TN, United States).

#### Subject population

The Correlating Outcomes with Biochemical Measurements to Estimate Time Progression in IPF study (COMET-IPF) (clinical trials ID no. NCT01071707) was a multi-center, prospective observational cohort aimed at identifying markers of IPF progression. All data and samples used in this study were de-identified. The study design has been described previously^8,26^, but in brief, eligible patients were aged 35–80 with a multidisciplinary IPF diagnosis (confirmed by clinical history, chest computed tomography (CT) scan, and a lung biopsy when necessary). Subjects with an IPF diagnosis > 4 years prior to screening, diagnosed collagen-vascular disorder, FEV1/FVC < 0.60, evidence of active infection at screening, or comorbid conditions likely to result in death within 1 year were excluded. Informed consent was obtained from all participating patients. Progression during an 80-week follow-up period was dichotomized by the composite occurrence of a relative decline in FVC of ≥ 10% or in the diffusion capacity of the lungs for carbon monoxide (DLCO) of > 15%, acute exacerbation, lung transplant, or death. Seventy-one patients were originally screened for inclusion in the COMET cohort, of which 60 were included in the analysis described here. Patients were excluded from analysis based on a lack of blood samples at all three time points or missing data such as DLCO or 6 min walk test as described in the original study^8^.

#### Sample acquisitions and measurements

Peripheral blood samples were collected from 60 COMET patients at three time points (week 0/baseline, week 48, and week 80). Slow off-rate modified aptamers (SOMAmer^©^) technology was used to measure 1,129 proteins present in blood samples at each collection time point. A small number of blood proteins in 15 of these samples were later also measured by ELISA; the concentrations of the two platforms were correlated using Pearson’s correlation coefficient.

Bronchoscopy was performed at enrollment in patients who were clinically stable and without evidence of active infection. Luminex FlexMAP 3D (Luminex Corporation, Austin, TX) technology was used to measure 29 cytokines/chemokines in the BAL samples. Samples below the lower limit of detection were set to be ½ the lowest minimum detectable concentration across the standard curves of all analytes. Before inclusion in any analyses, all BAL protein concentrations were normalized to total protein concentration as quantified by a Pierce BCA Protein Assay Kit (Pierce Protein Biology, Rockford, IL).

For more details on peripheral blood and BAL sample collection, please see the supplemental materials.

#### Data processing

Before beginning any analysis, a PCA model was created to identify potential negative drivers in the multivariate model. Negative drivers were defined as samples which disproportionally drove the final model such that model parameters solely explained the driver’s variance, and were characterized as samples with a Hotelling’s Reduced T^2^ statistic value > 5. The sample with the highest Hotelling's Reduced T^2^ statistic greater than 5 was subsequently removed and another PCA model was generated based on the remaining data. This process was iteratively implemented until all samples produced Hotelling’s Reduced T^2^ statistics < 5, resulting in 4 unique datasets with the following features: (1) baseline blood proteins (59 samples; 34 progressors and 25 non-progressors; demographics detailed in Supplemental Table S1), (2) BAL proteins (51 samples; 31 progressors and 20 non-progressors; demographics in Supplemental Table S2), (3) baseline blood and BAL proteins (50 samples; 30 progressors and 20 non-progressors; demographics in Table 1), (4) temporal-dependent blood proteins for trajectory PCA (102 progressor and 71 non-progressor time point measurements in total). The associated univariate analyses contained the same spread of samples.

All proteins, both those measured by SOMAmers^©^ and by Luminex, were measured in both progressors and non-progressors and included in the initial LASSO analysis.

#### Statistical analysis of differential protein expression in clinical cohorts

Two volcano plots illustrated individual blood and BAL proteins that were significantly and differentially expressed across IPF progressors and non-progressors. Relative fold-changes in blood and BAL protein levels were calculated by dividing the average expression of each protein in progressors by that in non-progressors. Statistical analysis between protein expression in the cohorts was performed by standard two-sample t-tests. P-values < 0.05 were regarded as significant.

#### Identification of proteomic signatures with feature selection tools and PLSDA

PLSDA was used in conjunction with feature selection tools to determine the protein signature which best differentiated clinical cohorts in various datasets. Prior to any analysis, data were normalized with mean centering and variance scaling. The LASSO was used when finding the minimum signature based on SOMAmer^©^ blood protein data. For all LASSO models, k-fold cross-validation (k = 10) was used to generate the model with the lowest possible mean squared error for prediction, such that random subsets were iteratively excluded from the data set during model calibration and were later used to evaluate model predictions. Variable importance in projection (VIP) scores identified the differentiating signature of BAL proteins, with a VIP cutoff score for inclusion in the model of ≥ 1. All PLSDA models were built using k-fold cross-validation (k = 10) and were orthogonalized to improve interpretability. ROC curves were generated based on the classification ability of a PLSDA model.

#### Analysis of differentially expressed proteome with DAVID

The Database for Annotation, Visualization, and Integrated Discovery (DAVID) was used to identify significantly enriched biological processes based on the protein signatures identified by multivariate methods. Protein signatures which resulted from these approaches were sorted into profiles based on their relative expression levels in progressor or non-progressor cohorts. The sign of the PLSDA loadings on LV1 determined if the protein was comparatively increased in progressors (negative loadings) or non-progressors (positive loadings). The resulting clustering and enrichment diagrams from DAVID were created by searching through Gene Ontology (GO) biological processes (BP FAT), GO molecular function (MF FAT), and Kyoto Encyclopedia of Genes and Genomes (KEGG). Only the clusters and pathways which were significant after applying the Bonferroni correction within DAVID were reported.

#### Comparison of PLSDA model performance parameters

In order to quantitatively compare calibration accuracy across multiple PLSDA models, each model of interest was probed to determine whether it correctly or incorrectly classified each individual patient. Patients who were not included in all of the models to be compared were unable to be included in this comparative analysis of calibration accuracy, which only affected the comparison of models based on multiple tissue compartments. A matrix of matched sets of proportions was generated where each patient’s classification state (e.g. correctly or incorrectly classified by the model) was represented as dichotomous values for each of the models of interest. These proportions were then compared using Cochran’s Q test in conjunction with McNemar’s post hoc test; significance was defined as the adjusted *p* < 0.05.

To compare cross-validation accuracy between models, we split the total data into ten groups (5–6 samples in each group) and then iteratively generated PLSDA models based on nine groups the data, and tested the model with the unused group of data. We recorded if these test samples were accurately classified by the model, and compared the percent accuracy from all ten groups associated with one model to percent accuracy of other models. Statistical significance between models was evaluated by a standard one-way ANOVA with Tukey’s post hoc test. *P*-values < 0.05 were deemed significant.

#### Visualization of classification ability of LASSO-identified signature using clustering

Hierarchical clustering of the LASSO-identified signature based on blood and BAL proteins was generated with supervised average linkage clustering. Pearson’s correlation coefficient was used as the distance metric. Samples were colored by progression status as well as other clinical, radiologic, and genetic variables.

#### Exploration of network interactions between progressor and non-progressor cohorts

Protein correlation networks were constructed separately for progressors and non-progressors using pairwise Pearson’s correlation coefficients between protein expression in the LASSO-identified signature within the two groups. Edge color and thickness correspond to coefficient value and statistical significance, respectively, with only significant correlations (*p* < 0.05) being shown. Node size is proportional to its degree of connectedness.

#### Investigating temporal dependences on progressor/non-progressor protein signatures

LASSO identified the minimum blood signature that differentiated the three collection time points (week 0, 48, and 80) in progressors and non-progressors separately. Trajectory PCA models^33^ were then created based on each of these signatures. A one-way ANOVA with Tukey’s post hoc test was used to evaluate the significance of temporal differences in protein expression by comparing the scores on PC1 at each collection time point. P-values < 0.05 were considered significant.

#### Visualization of time-dependent scores with density plots

PC1 scores from each of the three time points in the trajectory PCA were fit to a kernel distribution. The kernel distribution was reconstructed into a probability density function using the *fitdist* function with the normal smoothing function and the default bandwidth value.

#### Software summary

All volcano plots, hierarchical clustering, heat maps, correlation networks, and density plots were completed using MATLAB (v2016b, MathWorks, Natick, MA). LASSO was implemented using MATLAB software^39^. PCA and PLSDA models, ROC curves, and VIP score calculations were generated using the PLS toolbox available in MATLAB (v8.2.1, Eigenvector, Mason, WA). All statistics, with the exception of Cochran’s Q test, were performed using Prism version 7.00 and version 8.00 (GraphPad software, San Diego, CA). Cochran’s Q test with McNemar’s post hoc test was done in R software version 3.5.1 (R Core Team, Vienna, Austria).

## Supplementary information

Supplementary data

Supplementary information

## Data Availability

The SomaLogic blood protein measurements were previously published by Ashley et al.^20^, and are also available at this link: https://sites.google.com/a/umich.edu/bethany-b-moore-lung-immunobiology-lab/home/datasets-from-our-publications. The BAL Luminex data is available in the supplementary materials.
