# Supplementary material for: Identification of a unique temporal signature in blood and BAL associated with IPF progression
Source: Sci Rep. 2020 Jul 21;10:12049. doi: 10.1038/s41598-020-67956-w (PMC7374599; doi:10.1038/s41598-020-67956-w)
Supplement: Supplementary file 2 — Supplementary information [file 41598_2020_67956_MOESM2_ESM.docx]

**Identification of a unique temporal signature in blood and BAL associated with IPF progression**

Katy C. Norman^1^, David N. O’Dwyer^2^, Margaret L. Salisbury^3^, Katarina M. DiLillo^1^, Vibha N. Lama^2^, Meng Xia^4^, Stephen J. Gurczynski^2^, Eric S. White^2^, Kevin R. Flaherty^2^, Fernando J. Martinez^5^, Susan Murray^4^, Bethany B. Moore^2,6^, and Kelly B. Arnold^1^

**Methods**

**Sample acquisitions and measurements**. Peripheral blood samples were collected from 60 COMET patients at three time points (week 0/baseline, week 48 and week 80) in EDTA-containing vacutainers and were shipped overnight from individual sites to the University of Michigan. Blood samples were centrifuged and plasma was stored at -80°C until transported to SomaLogic (Boulder, CO). Slow off-rate modified aptamers (SOMAmer^©^) technology was used to measure 1129 proteins present in blood samples at each collection time point.

Bronchoscopy was performed at enrollment in patients who were clinically stable and without evidence of active infection. BAL samples were collected and pooled from 4 installations of 50 mL sterile isotonic saline aliquots. Cell-free fluid was stored at -80°C. Luminex FlexMAP 3D (Luminex Corporation, Austin, TX) technology was used to measure 29 cytokines/chemokines in the BAL samples. Samples below the lower limit of detection were set to be ½ the lowest minimum detectable concentration across the standard curves of all analytes. Before inclusion in any analyses, all BAL protein concentrations were normalized to total protein concentration as quantified by a Pierce BCA Protein Assay Kit (Pierce Protein Biology, Rockford, IL).

**Table S1.** Demographic and lung function test descriptions from progressors and non-progressors whose baseline blood protein measurements were used in creating models based on blood proteins alone.

|  | **Non-progressor (N=25)** | **Progressor (N=34)** | **P-value** |
| --- | --- | --- | --- |
| Age | 63.72 | 64.86 | 0.5855 |
| Sex (Male) | 76% | 61.76% | 0.2551 |
| Number Never Smokers | 7 | 12 | 0.5614 |
| Number Former Smokers | 17 | 22 | 0.796 |
| Number Current Smokers | 1 | 0 | 0.2469 |
| FVC % Predicted | 68.19 | 70.78 | 0.5511 |
| DLCO % Predicted | 44.75 | 47.61 | 0.441 |

**Table S2.** Demographic and lung function test descriptions from progressors and non-progressors whose baseline BAL protein measurements were used in creating models based on BAL proteins alone.

|  | **Non-progressor (N=20)** | **Progressor (N=31)** | **P-value** |
| --- | --- | --- | --- |
| Age | 62.43 | 65.43 | 0.1924 |
| Sex (Male) | 16 (80%) | 20 (64.5%) | 0.2446 |
| Number Never Smokers | 6 (30%) | 11 (35.48%) | 0.6922 |
| Number Former Smokers | 13 (65%) | 20 (65.42%) | 0.9725 |
| Number Current Smokers | 1 (5%) | 0 | 0.2165 |
| FVC % Predicted | 66.88% | 71.84% | 0.3248 |
| DLCO % Predicted | 45.78% | 47.42% | 0.6803 |

**Table S3.** Pearson’s correlation between proteins measured by SOMAmer aptamers and by ELISA in a subset of the COMET samples.

|  | Pearson's correlation coefficient | P-value |
| --- | --- | --- |
| CCL22 | 0.672 | 0.006 |
| CCL18 | 0.706 | 0.003 |
| CCL2 | 0.566 | 0.028 |
| IL-10 | -0.208 | 0.456 |
| CXCL12 | -0.081 | 0.775 |


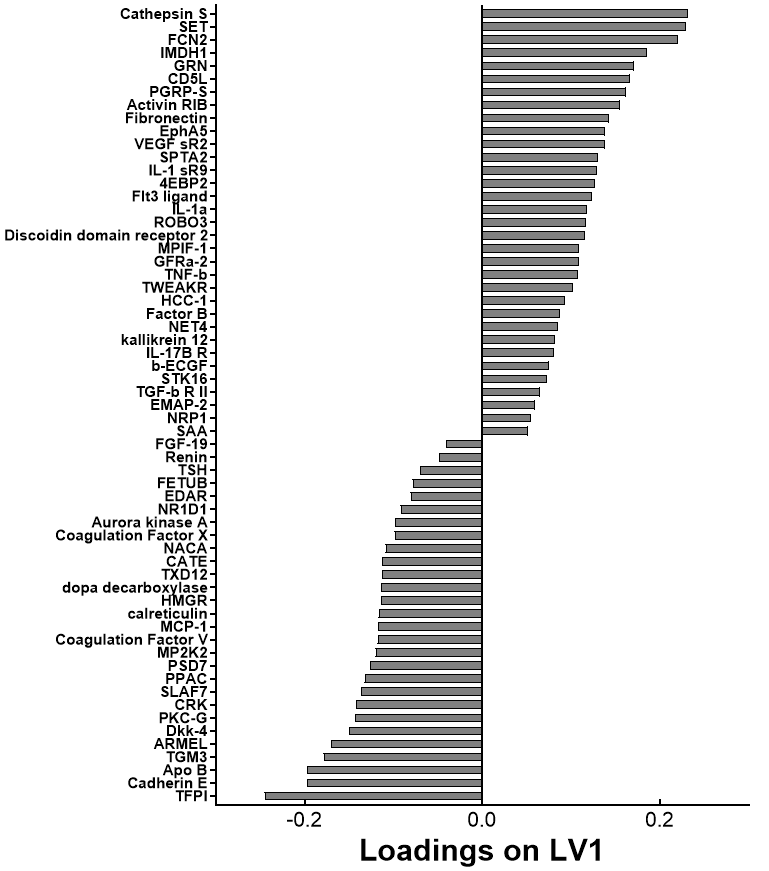

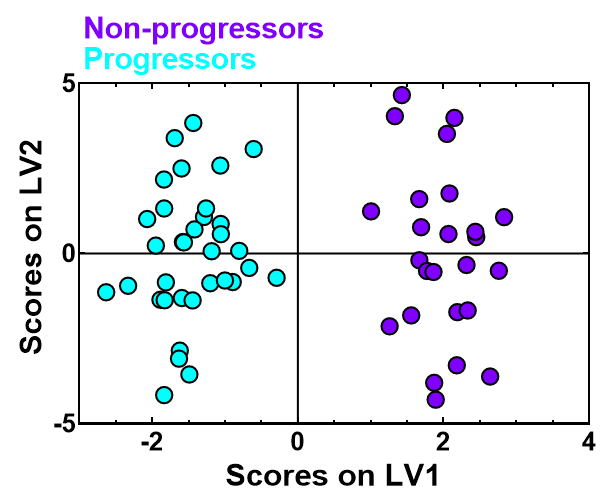


b

a

**Figure S1.** The PLSDA model based on LASSO-identified signature of blood proteins is accurately able to differentiate IPF progressors and non-progressors. **(a)** LASSO identified a signature of 61 blood proteins that differentiated progressors and non-progressors with 100% calibration and 96.53% cross-validation accuracy. **(b)** The associated loadings on latent variable 1 (LV1) captured 6.28% of the total variance in the data. Proteins that are loaded negatively on LV1 are comparatively upregulated in IPF progressors, and positively loaded proteins have a comparative reduction in IPF progressors.


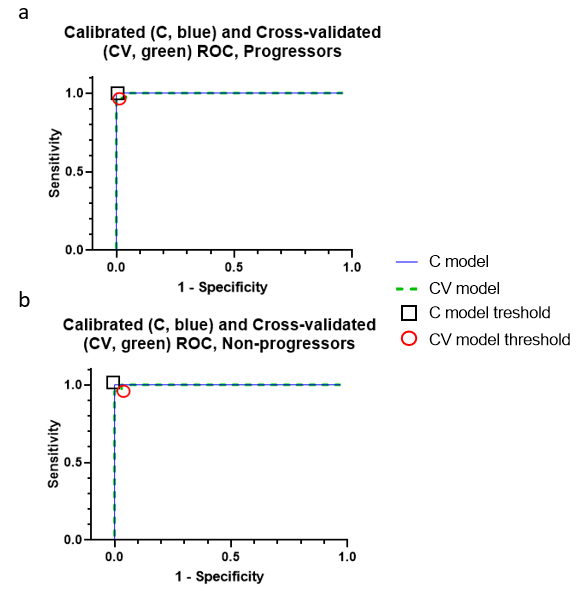


**Figure S2.** The receiver operator characteristic (ROC) curves associated with the PLSDA model based on the LASSO-identified signature of 61 blood proteins. **(a)** The cross-validated PLSDA model reported a sensitivity of 97.06% and a specificity of 99.56% for the progressors. **(b)** The cross-validated PLSDA model reported a sensitivity of 96% and specificity of 97.38% for the non-progressors. C: Calibrated; CV = Cross-validated; AUC = area under curve.


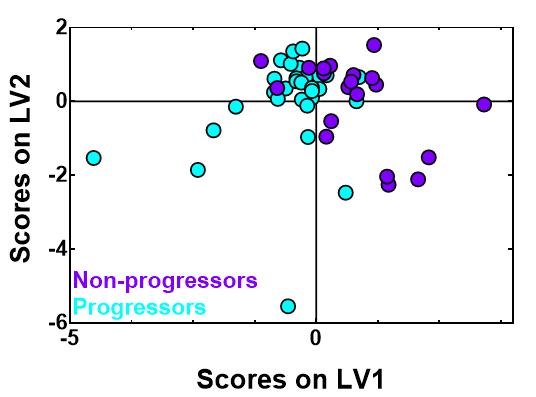

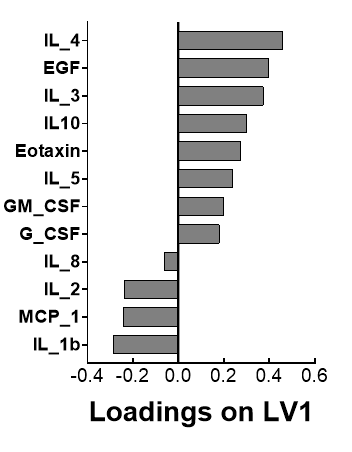


b

a

**Figure S3.** PLSDA model based on VIP-selected signature of BAL proteins is moderately able to differentiate IPF progressors and non-progressors, with 78.55% calibration and 67.82% cross-validation accuracy. **(a)** The PLSDA scores plot of the 12 feature BAL protein signature highlights moderate separation between baseline progressors and non-progressors, with progressors generally having negative scores on LV1 and non-progressors having positive scores. **(b)** The associated loadings on LV1 captured 16.49% of the total variance in the data. Proteins that are loaded negatively on LV1 are comparatively upregulated in IPF progressors, and positively loaded proteins have a comparative reduction in IPF progressors.


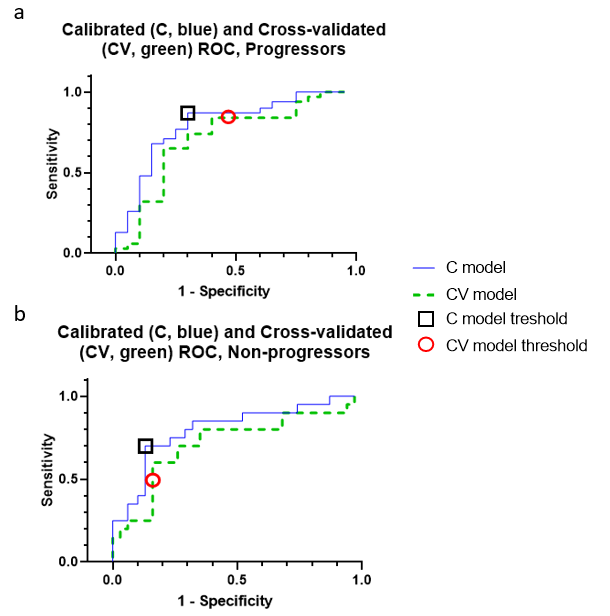


**Figure S4.** The receiver operator characteristic (ROC) curves associated with the PLSDA model based on the VIP-selected signature of BAL proteins. **(a)** The cross-validated PLSDA model reported a sensitivity of 83.87% and a specificity of 54.94% for the progressors. **(b)** The cross-validated PLSDA model reported a sensitivity of 49.94% and specificity of 83.87% for the non-progressors. C: Calibrated; CV = Cross-validated; AUC = area under curve.

**Figure S5**

**Figure S5 continued**

**Figure S5 continued**


**Supplemental Figure S5.** Direct comparison of expression of the blood and BAL proteins in the LASSO-identified signature in both progressors and non-progressors. Significance according to a two-sample t-test is marked on each graph, with ^**^ indicating p < 0.01 and ^*^ indicating p < 0.05.


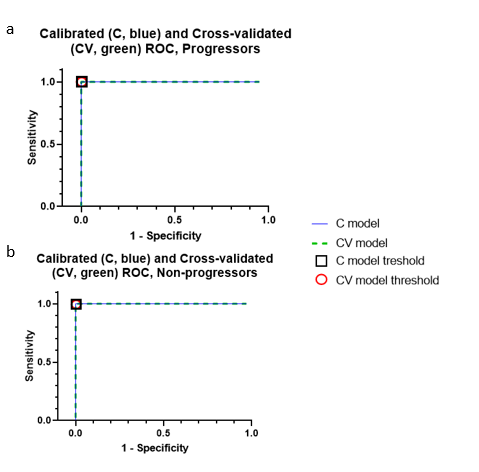


**Figure S6.** The receiver operator characteristic (ROC) curves associated with the PLSDA model based on the LASSO-identified signature of blood BAL proteins. **(a)** The cross-validated PLSDA model reported a sensitivity of 100% and a specificity of 100% for the progressors. **(b)** The cross-validated PLSDA model reported a sensitivity of 100% and specificity of 100% for the non-progressors. C: Calibrated; CV = Cross-validated; AUC = area under curve.


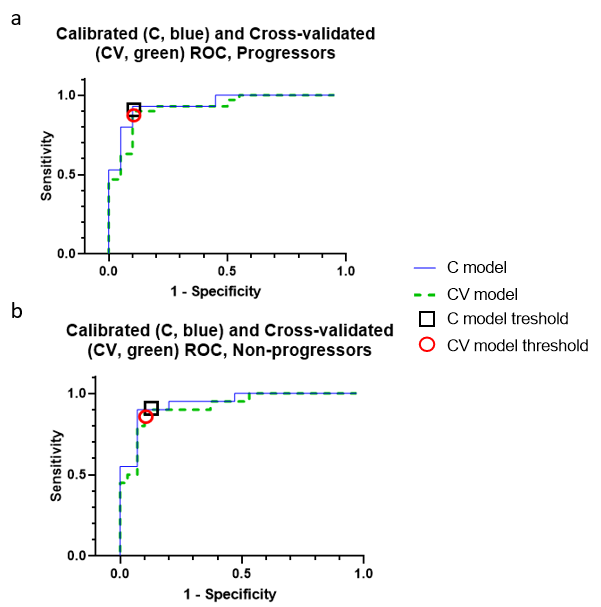


**Figure S7.** The receiver operator characteristic (ROC) curves associated with the PLSDA model based on the 28 proteins that were identified as being significantly differentially expressed across progressors and non-progressors in the volcano plot. **(a)** The cross-validated PLSDA model reported a sensitivity of 88.29% and a specificity of 87.56% for the progressors. **(b)** The cross-validated PLSDA model reported a sensitivity of 90% and specificity of 90% for the non-progressors. C: Calibrated; CV = Cross-validated; AUC = area under curve.


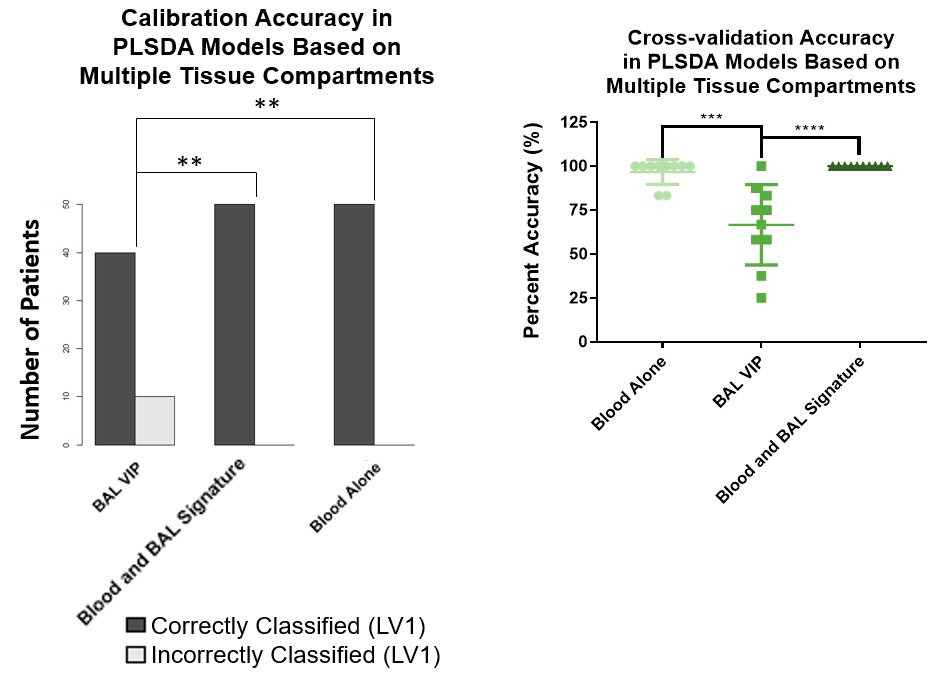


a

b

**Figure S8.** Comparison of calibration and cross-validation accuracy in the PLSDA models based on blood proteins alone, BAL proteins alone, and blood and BAL proteins combined shows that the blood only and the combination model are both significantly better than the model based on BAL proteins alone. **A.** Comparison of the calibration accuracies in the three PLSDA models. ** indicate p < 0.01 after administration of Cochran’s Q test with McNemar’s post hoc test. **B.** Comparison of the cross-validation accuracies of the same three PLSDA models shown in panel **A**. *** indicates p = 0.0001, and **** indicates p < 0.0001 after administration of a one-way ANOVA with Tukey’s multiple comparison test.


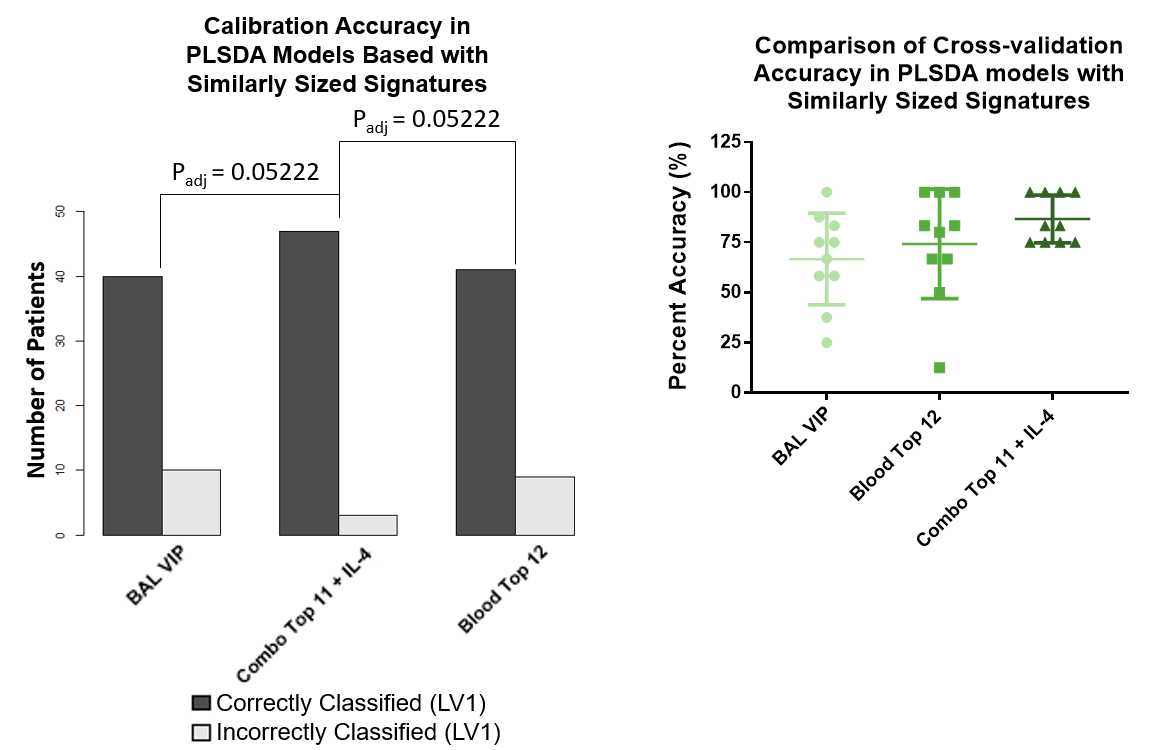


b

a

**Figure S9.** Statistical comparison of calibration and cross-validation accuracies of PLSDA models with similar number of features included in each signature showed only trends towards being significantly different from each other. **A.** Statistical analysis of the calibration accuracies via Cochran’s Q test showed that the shortened signature based on blood and BAL proteins combined approached being significantly better than the BAL VIP and the shortened blood signature (p = 0.052, McNemar’s post hoc test). **B.** When comparing cross-validation accuracies of the three models, none were significantly different from each other.

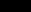


Indicates involvement in process

Indicates no involvement in process

a

b

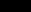


Indicates involvement in process

Indicates no involvement in process

**Figure S10.** Additional DAVID enrichment analyses of the proteins in the LASSO-signature that were found to be comparatively upregulated in the non-progressors. **A.** This cluster was mostly enriched for processes involving cell signaling and regulation of basic cell processes, with an enrichment score of 2.57. **B.** This cluster was also enriched for processes involving the function and regulation of the immune, defense and inflammatory responses, with an enrichment score of 2.50. Black squares indicate protein involvement in a particular pathway, while white squares indicate non-involvement.

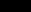


Indicates involvement in process

Indicates no involvement in process

**Figure S11.** DAVID enrichment analysis of the proteins that were comparatively upregulated in the progressors in the LASSO-identified signature based on blood and BAL proteins measured in COMET IPF patients. The enrichment score of this cluster is 2.05. Black squares indicate protein involvement in a particular pathway, while white squares indicate non-involvement.


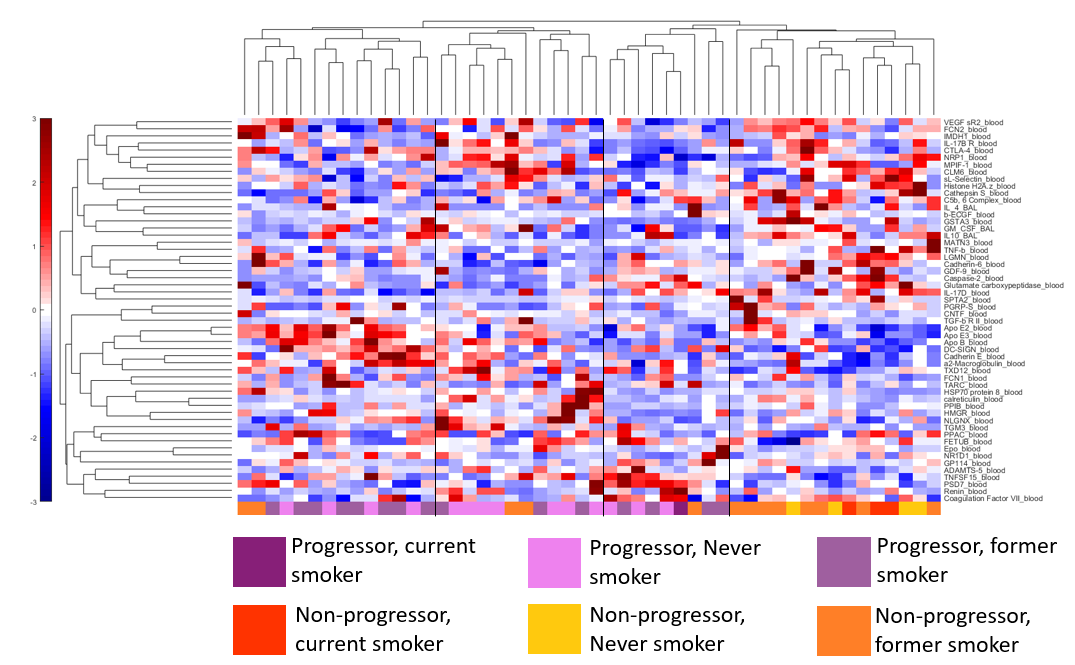


a


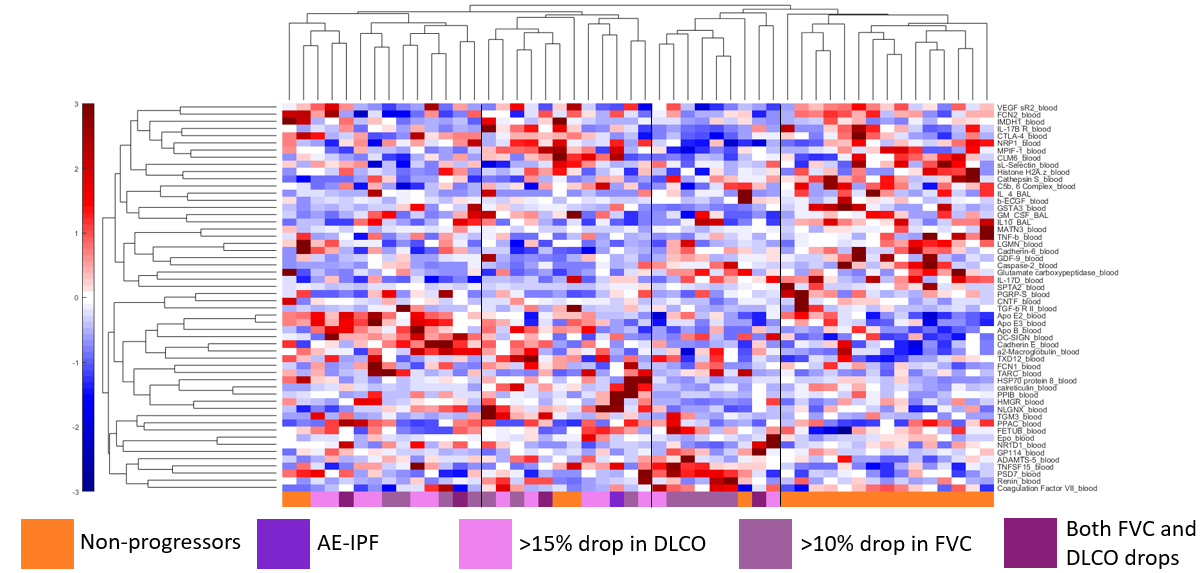


b


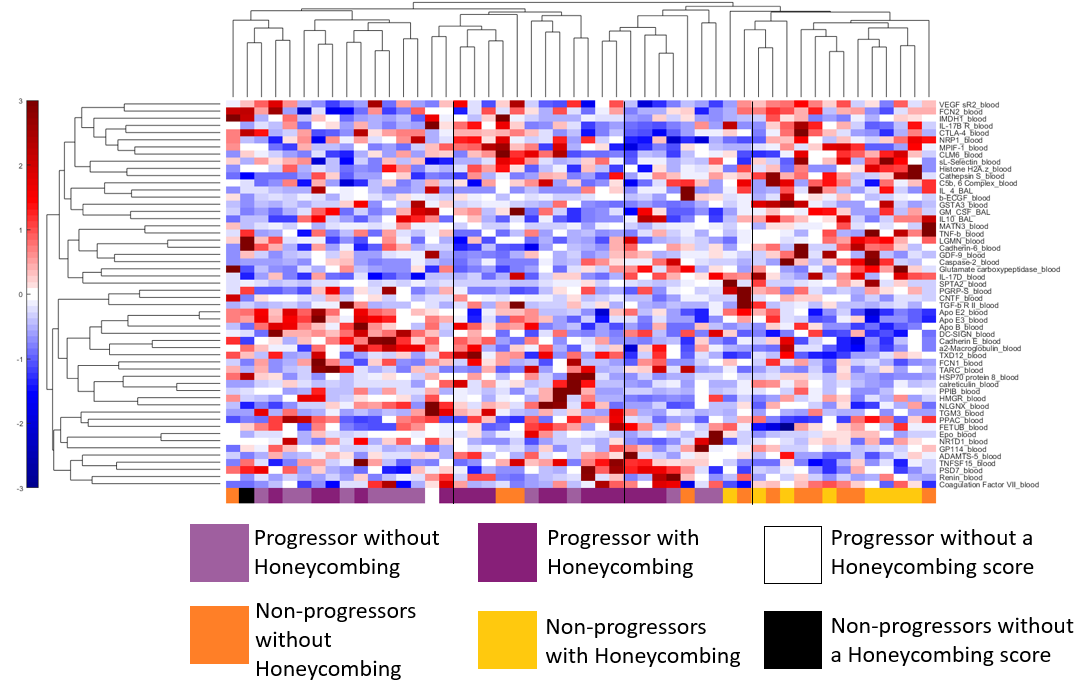


c


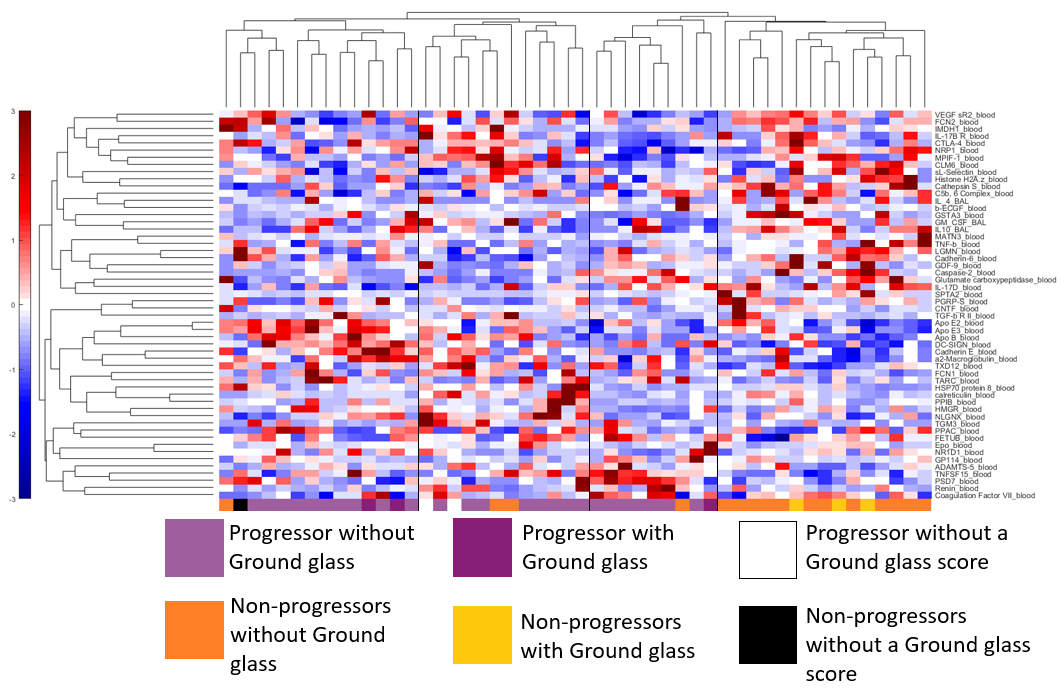


d

**
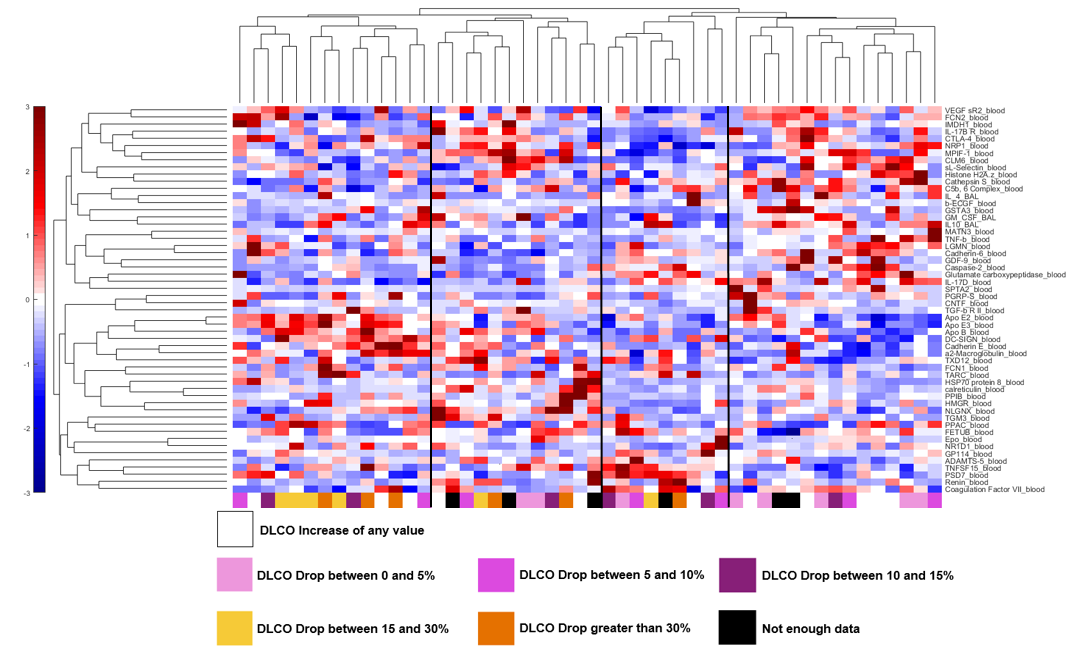

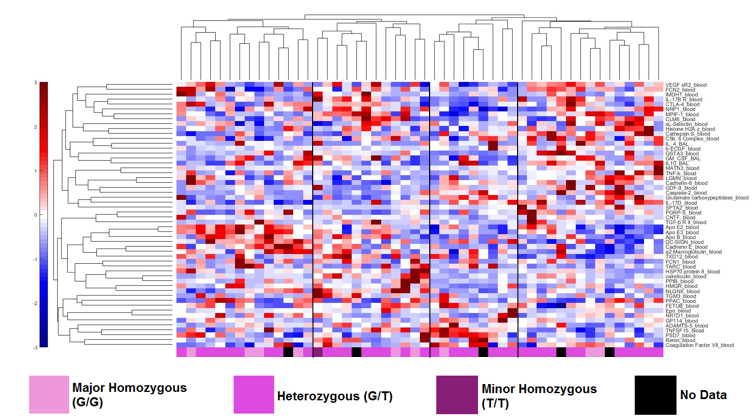
**

f

e

**Figure S12.** Hierarchical cluster based on the combination signature did not cluster according to the following clinical and pulmonary variables: **A.** smoking status, **B.** how progression occurred in that specific patient, **C.** presence of honeycombing in the CT scan, **D.** presence of ground glass in the CT scan, **E.** DLCO increase or decrease over the 80-week time period of the COMET study, **F.** MUC5b genotyping results, **G.** TOLLIP genotyping results, and **H.** MUC5b and TOLLIP genotyping results together. Color bars are shown to the left of each figure, with red indicating higher protein expression level from the mean, white unchanged, and blue a lower expression. AE-IPF: acute exacerbations of IPF, DLCO: diffusing capacity of the lungs for carbon monoxide, FVC: forced vital capacity.


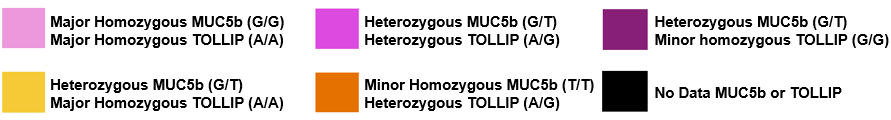

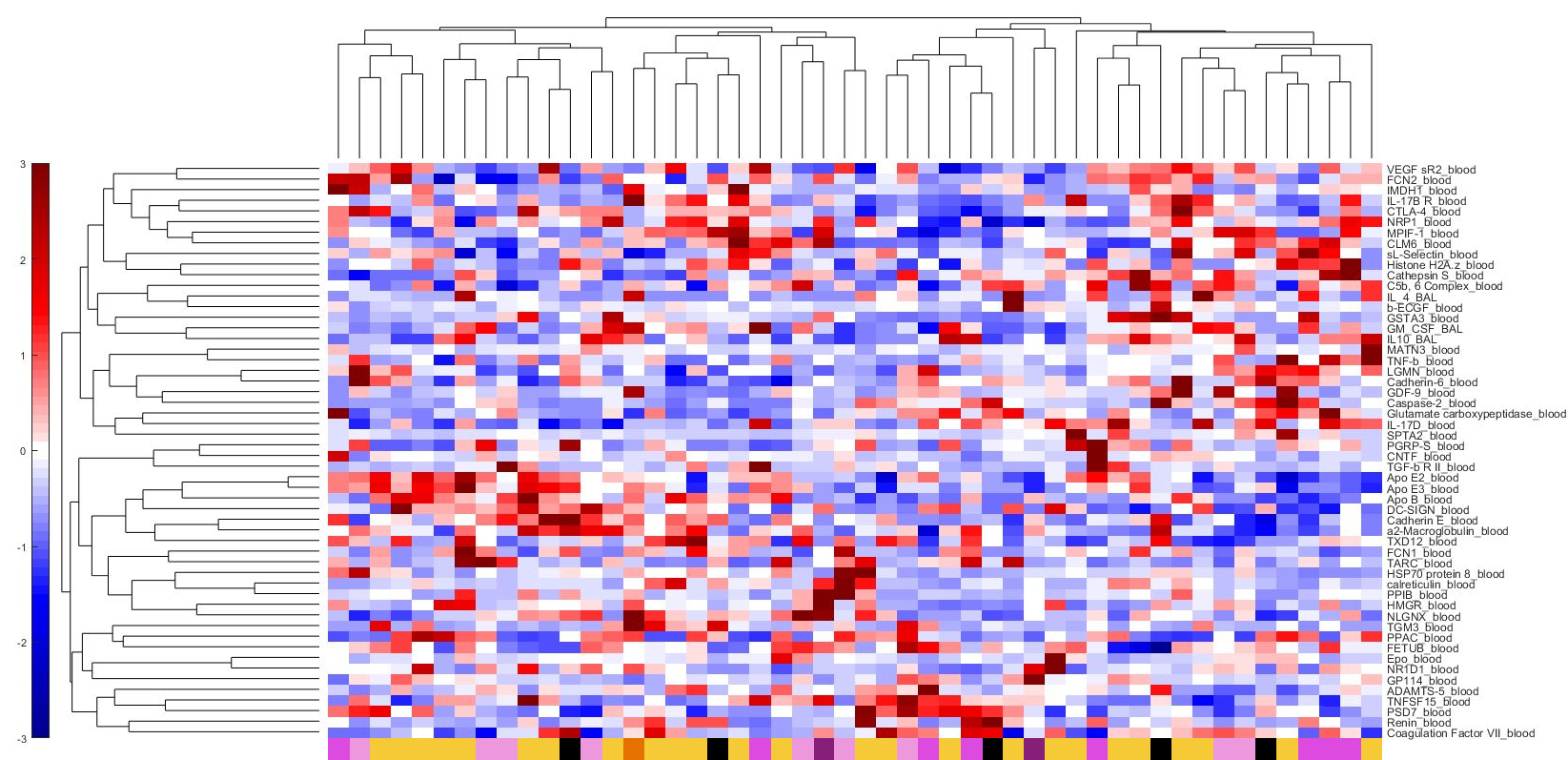


h


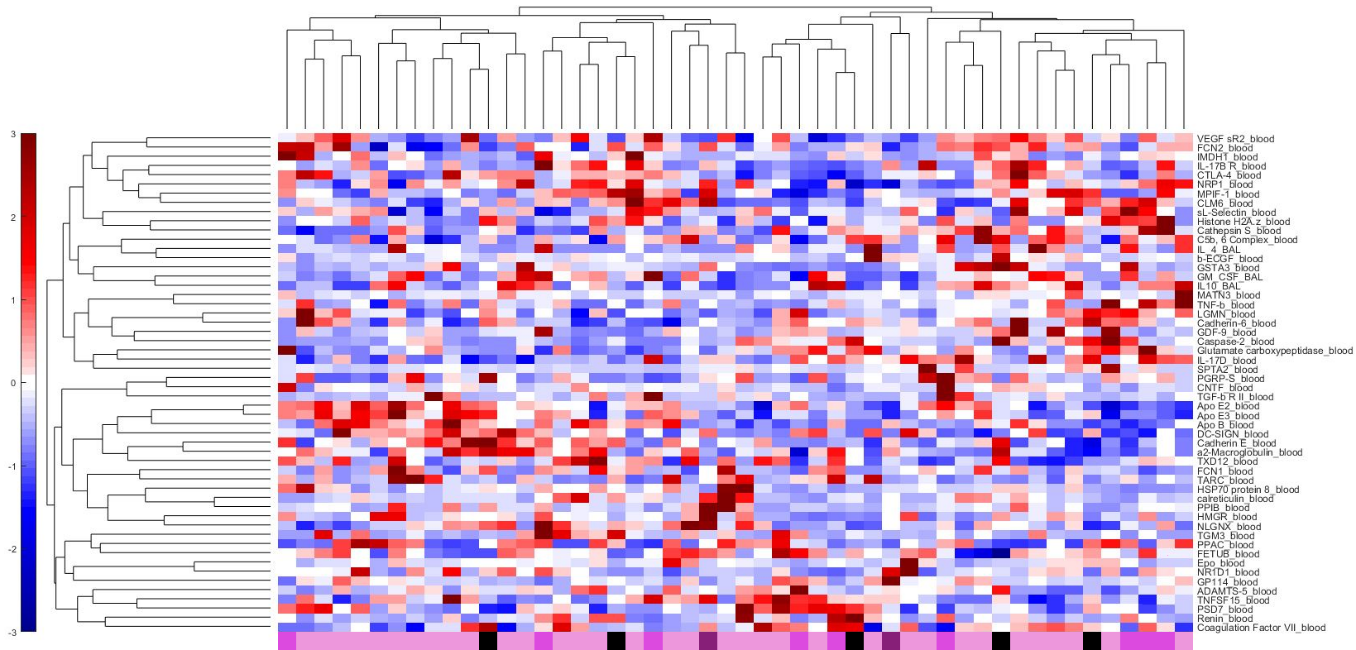

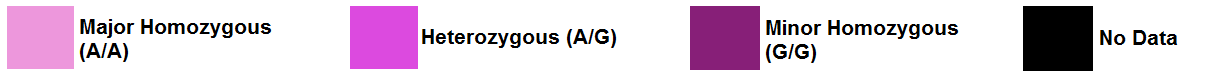


g

b


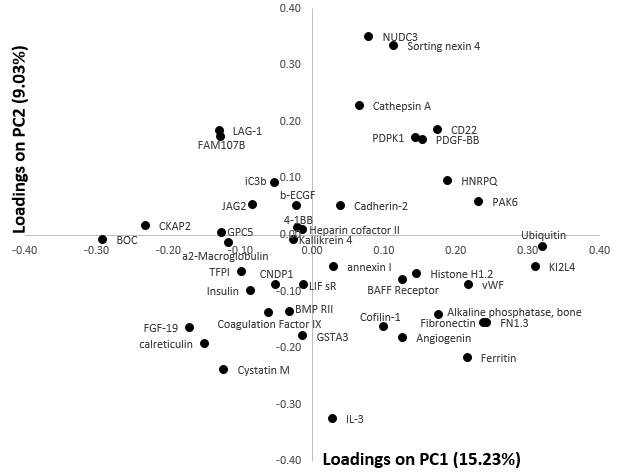


**Figure S13.** The LASSO-identified trajectory PCA signature chosen to separate the non-progressors across the three time points captured 24.26% of the natural variance in the data across the first two principal components.
